# Self‐Maintainable Electronic Materials with Skin‐Like Characteristics Enabled by Graphene‐PEDOT:PSS Fillers

**DOI:** 10.1002/advs.202410539

**Published:** 2025-04-25

**Authors:** Morteza Alehosseini, Firoz Babu Kadumudi, Sinziana Revesz, Parham Karimi Reikandeh, Jonas Rosager Henriksen, Tiberiu‐Gabriel Zsurzsan, Jon Spangenberg, Alireza Dolatshahi‐Pirouz

**Affiliations:** ^1^ Department of Health Technology Technical University of Denmark Kongens Lyngby 2800 Denmark; ^2^ Department of Electrical and Photonics Engineering Technical University of Denmark – DTU Kongens Lyngby 2800 Denmark; ^3^ Department of Civil and Mechanical Engineering Technical University of Denmark Kongens Lyngby 2800 Denmark

**Keywords:** adaptive robotics, bioelectronics, graphene‐PEDOT, self‐healing materials, soft electronics

## Abstract

Conventional devices lack the adaptability and responsiveness inherent in the design of nature. Therefore, they cannot autonomously maintain themselves in natural environments. This limitation is primarily because of using rigid and fragile material components for their construction, which hinders their ability to adapt and evolve in changing environments. Moreover, they often cannot self‐repair after injuries or significant damage. Even devices with self‐healing, soft, and responsive properties often fail to seamlessly integrate all these attributes into a single, scalable, and cohesive platform. In this study, a significant breakthrough is introduced by utilizing graphene‐poly(3,4‐ethylenedioxythiophene): polystyrene sulfonate (graphene‐PEDOT:PSS) fillers to transform a typically weak, insulating, and jelly‐like material into a soft electronic material with properties akin to those of living organisms, such as skin tissue. The developed electronic materials exhibit a range of other capabilities attributed to the hierarchical organization originating from filler enhancement, which includes methods such as heat regulation, 3D printability, and multiplex sensing. The introduction of this new class of materials can facilitate the self‐maintenance of life‐like soft robots and bioelectronics that can be seamlessly integrated within dynamic environments, such as the human body, while demonstrating the ability to sense, respond, and adapt to challenging environments.

## Graphical abstract

Graphene‐PEDOT:PSS fillers revolutionize soft electronics by transforming jelly‐like materials into skin‐like systems with self‐healing, heat‐responsiveness, and multiplex sensing capabilities. This novel material exhibits exceptional printability, flexibility, and adhesiveness, enabling seamless integration into bioelectronics and tissue‐cyborganic interfaces. Its life‐like properties make it ideal for wearables and dynamic environments, advancing applications in soft robotics and human machine interfacing.

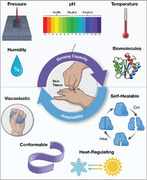

## 1 Introduction

Most existing electronic devices, such as robots, smart watches, computers, and mobile phones are fabricated using static, brittle, and rigid components because of the lack of commercially available adaptable counterparts with sufficient functionalities.^[^ ^1^, ^2^ ^]^ In recent years, efforts have been devoted to addressing the lack of technology by creating soft, tough, conformable, and self‐healing electronic material systems. This has increased the durability of electronic circuits, resulting in self‐healing and flexible electronic devices that can easily blend in with the curved and soft anatomy of humans.^[^ ^1^, ^3^, ^4^, ^5^, ^6^ ^]^ Despite their advantages, soft electronic materials continue to display several shortcomings that limit their use in the real world. This is because of the trade‐offs between elasticity, material strength, and self‐healing time. For instance, materials that can autonomously self‐heal within seconds without requiring external stimuli are highly viscous, with low strain recovery and suboptimal mechanical integrity.^[^ ^5^, ^7^, ^8^ ^]^ This significantly weakens their field applications, where their reparation is compromised or not possible, and the available avenues for external stimuli are almost non‐existent.

Recent developments in developing soft materials with the incorporation of tannic acid (TA), graphene, poly(3,4‐ethylenedioxythiophene):polystyrene sulfonate (PEDOT:PSS) have introduced promising solutions to these limitations.^[^ ^6^, ^7^, ^9^, ^10^ ^]^ Composite films combining PEDOT:PSS with components such as ethylene glycol and polyvinyl alcohol have demonstrated a unique balance of high adhesion, stretchability, and self‐healing properties, making them suitable for both wearable and implantable bioelectronics.^[^ ^9^, ^10^, ^11^ ^]^ These materials provide improved electro‐mechanical properties and biocompatibility, as shown in applications ranging from biopotential monitoring to neural stimulation.^[^ ^12^ ^]^

The aforementioned material systems maintain themselves only by healing their injured parts. For instance, they are not capable of sensing, responding, or adapting to their surroundings, which is a prerequisite for the self‐maintenance and self‐perseverance of natural organisms. Typically, living organisms use a self‐healing outer skin surface that can sense and respond to their surroundings to remain alive in the wild. To function appropriately over prolonged periods, medical wearables, machines, and robots must feel and respond to their environments rapidly and reliably, similar to natural living systems. Engineers have achieved this by incorporating expensive, rigid, heavy, and bulky silicon‐based electronics into soft robotic systems, bionic implants, wearables, and smart consumer products.^[^ ^3^, ^13^ ^]^ However, in addition to exhibiting self‐healing abilities, a single cohesive material platform that can sense, adapt, and respond to a broad spectrum of environmental factors via its biological, physical, and electronic properties should be developed in a cost‐effective, straightforward, and scalable manner, similar to the naturally existing living biological systems.

**Figure 1:**
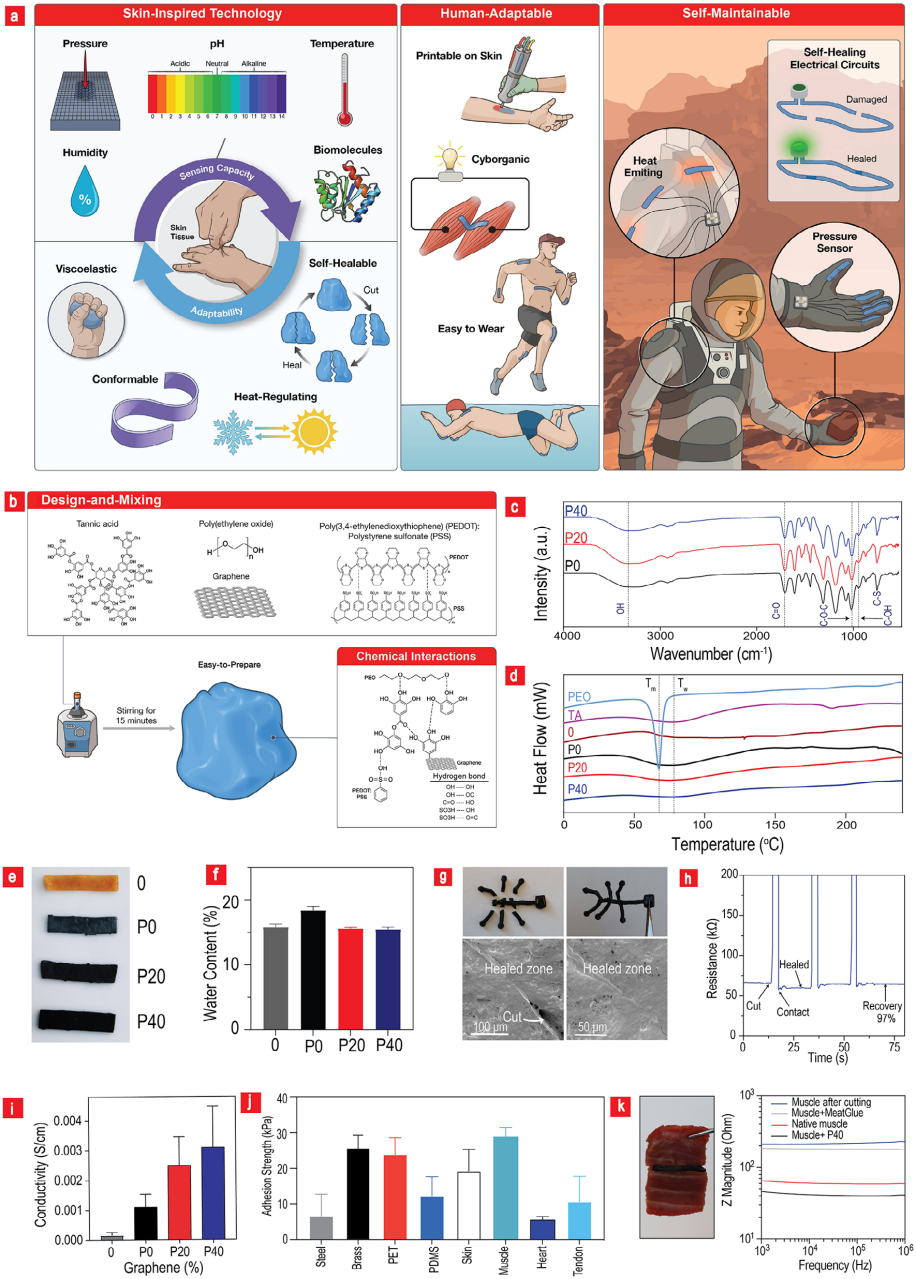
**Concepts and properties of the materials**. a) Conceptual illustration of the various applications of the material. b) Schematic of the fabrication process and the underlying chemistry. c) Fourier transform infrared (FTIR) spectra of synthesized materials. d) DSC profiles assessing the intrinsic polymeric structure of the materials (n = 3). e) Photographs of the as‐prepared samples. f) Quantification of water content in the samples as a function of filler concentration (n = 8). g) Efficient reassembly of the components after being torn apart. SEM images presenting a detailed examination of the rapid self‐healing property inherent in **P40** (n = 3). h) A cyclic demonstration of the spontaneous healing of the electronic circuit. The approximate recovery time for each cycle is 20 s (n = 4). i) Relationship between added fillers and conductivity (n = 5). j) Quantified adhesion strength on steel, brass, PET, polydimethylsiloxane (PDMS), skin, muscle, heart, and tendon, further emphasizing the versatility of **P40** as an adhesive. k) Ability of **P40** to heal broken muscle tissue both mechanically and electrically (n = 4). All data are presented as mean ± SD.

Herein, we used graphene poly (3,4‐ethylenedioxythiophene):polystyrene sulfonate (graphene‐PEDOT:PSS) fillers to impart such life‐like mechanical and electronic properties to an otherwise non‐functional and fragile material. Mechanical enhancement increases durability, whereas electronic endowment enables materials to sense and respond to various environmental cues. In particular, this new soft electronic material system exhibits a wide range of properties, including self‐healing ability, skin‐like softness, heat‐responsiveness, printability, adhesiveness to almost any surface type, and flexibility, while displaying excellent sensing capacity across the domains of biology, physics, and chemistry (**Figure** **1**a). The proposed skin‐like material system can easily conform and adhere to the human body, identical to its natural counterpart; therefore, it can be ideal for use in tissue–cyborganic interfaces and as a component for future wearables (Figure 1a). We used a simple, scalable, and non‐toxic methodology to realize the aforementioned features. Poly(ethylene oxide) (PEO) was mixed with PEDOT:PSS, reduced graphene oxide (rGO), and TA, and the mixture was vortexed for 15 min to facilitate the manufacture of the material (Figure 1b). The material self‐healed within seconds while displaying complete recovery up to almost 600% strain values, with stability over 10 000 cycles and Young's modulus values in the sub‐MPa range. Moreover, the combination of fillers induced certain electrical, thermal, and electrochemical properties, which were used to generate a heat‐regulating material capable of physical, chemical, and biological sensing. Furthermore, the system sensed pressure, strain, pH, humidity, temperature, dopamine, hydrogen peroxide, ascorbic acid, and bovine serum albumin (BSA) using only one sensor (Figure 1a).

To the best of our knowledge, the developed material is the only synthetic material that exhibits multiple features, including flexibility, adaptability, self‐healing capability, adequate heating capacity, printability, and an unmatched sensing portfolio owing to its electronic properties. We used a novel 3D printing technique to demonstrate these unique advantages in real‐life applications. The developed materials were printed in a suspension bath to enable 3D complex curvilinear prints on robots, which has not been realized in the field to date. Notably, the electronic circuit was 100% functional, conformable, and self‐healable. Moreover, the robot could sense and respond to obstacles. Furthermore, we generated a 3D‐printed skin bandage for healthcare monitoring of a wide range of physical and biochemical disease markers. These 3D printable electronic materials are suitable for use as on‐site printable synthetic skins on either humans or robots to expand their perception and capacity to interact with the surrounding world. In addition to such health‐related applications, this novel artificial skin interface can facilitate the development of self‐repairing, pressure‐responsive, and heat‐regulating components that can be used in outer space and other challenging environments, wherein access to repair facilities and the comfort of civilization is limited. For instance, when combined with pressure and strain, the proposed material can potentially be used to create intelligent and self‐maintaining space suits that can regulate heat and provide haptic feedback from the environment (Figure 1a). Therefore, the developed self‐maintainable electronic material is highly valuable and has a wide range of applications in multiple fields.

## 2 Results and Discussion

To achieve a high level of multifunctionality with minimal trade‐offs, we combined various components, each serving a distinct purpose. PEO was used to provide structural integrity and high elasticity to the material, whereas PEDOT:PSS and rGO were incorporated to introduce additional electrical and electrochemical functionalities. Additionally, the inclusion of a star‐shaped molecule, namely TA, with hydroxyl, pyrogallol, and catechol groups further enhanced the properties of the material, imparting self‐healing, 3D printing, and adhesion capabilities to the final combinations (Figure 1b). We fabricated materials **0** (PEO‐TA), **P0** (PEO‐TA‐PEDOT:PSS), **P20** (PEO‐TA‐PEDOT:PSS‐20%GO), and **P40** (PEO‐TA‐PEDOT:PSS‐40%GO). Owing to the inclusion of GO and PEDOT:PSS, the color of the material transitioned from brownish to blackish while maintaining a water content of ≈15% (Figure 1e,f). The water content was slightly higher for **P0** because of the presence of the hydrophilic PSS backbone; however, it decreased with increasing amounts of hydrophobic rGO. By contrast, the porosity increased slightly after GO inclusion (Figure S1a, Supporting Information). The resulting material demonstrated an impressive portfolio of properties, including high stretchability, conductivity, adhesiveness, biophysical and chemical sensing capabilities, rapid self‐healing, printability, and adaptability to the human body. In the subsequent sections, we explore the electrical, chemical, and mechanical characterizations to better understand the underlying mechanisms responsible for this wide range of abilities.

### 2.1 Chemical Characterization

The chemical interactions resulting in the developed electronic materials were examined using Fourier‐transform infrared (FTIR) spectroscopy. As depicted in Figure S1b,c, (Supporting Information) the peak‐shifts in the FTIR spectra demonstrate a cross‐linking scheme, primarily including hydrogen bond interactions such as OH….OH (4.7 kcal mol^−1^), C‐O‐C…. HO (5.02 kcal mol^−1^), and C = O…HO (4.6 kcal mol^−1^) between TA and PEO, within TA and PEO themselves,^[^ ^14^, ^15^ ^]^ and interactions with PEDOT:PSS molecules via OH and C = O groups available on TA and aromatic sulfonate groups present in PEDOT:PSS, including the SO_3_H…HO (3–4 kcal mol^−1^) and SO_3_H…O = C hydrogen bonds.^[^ ^11^, ^14^ ^]^ In addition to FTIR spectroscopy, we performed X‐ray diffraction analysis to demonstrate the presence of graphene sheets within the composites. This was confirmed by the graphene planes corresponding to (002), which were clearly observed at a 2*θ* value of 20° in the composites (Figure S1c, Supporting Information). Additionally, we determined a 2*θ* value of 11°, indicating stacking of graphene layers owing to the intercalation of TA.^[^ ^16^ ^]^

### 2.2 Thermal Degradation Studies

All composites were subjected to thermogravimetric (TGA) and derivative thermogravimetric (DTG) analyses to determine their composition, molecular structure, and thermal stability (Figure S1d,e, Supporting Information). In particular, DTG enabled the identification of various important weight loss events corresponding to the minima and maxima in the TGA curves (Figure S1e, Supporting Information). Overall, the DTG results demonstrated that TA degraded first, followed by PEO degradation. This was reasonable considering the lower degradation temperature of TA compared with that of PEO. However, the maximum weight‐loss temperatures of TA and PEO decreased to a lower value in the composites. This implied that the semi‐crystalline structure of PEO, facilitated by strong intermolecular hydrogen bonds, was compromised in the composites owing to competitive hydrogen bonding with TA, PEDOT:PSS, and rGO, which resulted in a more amorphous polymeric matrix, thereby lowering the degradation temperature.

In addition to providing a deeper understanding of the molecular interactions in composites and their associated strengths, TGA measurements facilitated the determination of the approximate fraction of each compound in the composites. Based on the results depicted in Figure S1d,e (Supporting Information), we concluded that all compounds were present in the final compositions; the composition of PEO was constant at ≈40%, whereas that of TA was maximally at 60% with a slightly lower concentration after nanofiller inclusion. This indicates that hydrogen bonds in the form of OH…OH and CO…OH between TA and PEO were the dominating factors influencing the structural assembly inside the materials as they constituted most of the composite.

### 2.3 Crystallinity Studies

Differential scanning calorimetry (DSC) analyses were performed on pure PEO, TA powder, and the composites to gain a comprehensive understanding of their molecular structures and crystallinities (Figure 1d). The DSC results indicated that the crystallinity of PEO in the composites was completely disrupted, rendering them entirely amorphous. This was attributed to the hydrogen bonding interactions between TA and PEO, which are known to disturb the intramolecular links within the PEO backbone. The addition of PEDOT:PSS and rGO did not affect the amorphous nature of the composites. However, the specific melting enthalpy (ΔHm) of water evaporation temperature (Tw) decreased with the addition of PEDOT:PSS and rGO, primarily because of the hydrophobic nature of these components. In summary, the DSC analysis revealed that the composite assembly disrupted the crystalline nature of PEO owing to hydrogen bond interactions with TA, imparting the composites with exceptional stretchability and elasticity, as amorphousness served as a characteristic property in this context.

### 2.4 Intermolecular Interactions

The mechanical and structural integrity of the material system was determined by combining chemical and thermal analyses. The range of mechanical and self‐repair features discussed in the subsequent sections originates from simple hydrogen‐bonding chemistry based on a palette of different types of hydrogen bonds with varying strengths, ranging from 3 kcal mol^−1^ to 5.3 kcal mol^−1^, within an elastomeric framework of PEO. This is facilitated by the amorphous nature of the system resulting from its molecular interaction with TA. In this scenario, weak hydrogen bonds are reversible and thereby self‐healing. Therefore, they can open up an avenue for dissipating energy to bypass the stress build‐up and critical failure of the composite. However, the amorphous PEO framework endows the system with elastic integrity that would otherwise not be possible using hydrogen bonds alone. This resulted in a rapid self‐healing, highly stretchable, and tough system.

### 2.5 Self‐healing, Stretchability, Moldability, and Adhesion Studies

The proposed skin‐inspired electronic material is easily moldable, highly stretchable, and flexible despite being healed for a few minutes. For instance, the material can stretch up to 36 times its original length in a post‐healing scenario (Figure 1g; Figure S2.a,b, Supporting Information). We also used a Teflon mold to cast a complex self‐healing structure, which, after being torn apart, could rapidly reassemble while maintaining its flexibility (Figure 1g). The rapid self‐healing properties were examined using scanning electron microscopy (SEM; Figure 1g). The images illustrate the rapid restructuring of the materials within the defect zone, indicating that the material exhibits a major advantage over conventional self‐healing chemistries based on imine bonds, Diels–Alder reactions, and acylhydrazone bonds, as they typically require prolonged healing times, even exceeding 24 h.^[^ ^17^, ^18^ ^]^ Prolonged healing times are a major disadvantage because the material loses its electromechanical properties during the process, thereby requiring the implicated machinery to remain non‐operational for an entire day in a worst‐case scenario. The self‐healing properties observed in this study can be attributed to reversible and hierarchical hydrogen bonds, which are the primary driving forces behind the structural integrity of the composites. The observed low glass transition in Figure 1d, resembling that of a rubbery state, further enables material mobility during the healing process, thereby serving as a contributing factor to the observed low self‐healing time.

Owing to the high moldability, we developed a simple self‐healing electronic circuit connected to a light‐emitting diode (LED) lamp (Figure S2c, Supporting Information) in a Play‐Doh‐like manner, thereby obtaining a readily scalable and implementable circuit in almost any setting. The lamp was lit by applying an electrical voltage to the circuit, with the circuit healing immediately after enduring critical damage; this happened without the assistance of external stimuli. Figure 1h illustrates this phenomenon, wherein the circuit spontaneously heals in a cyclic manner with a recovery time of ≈20 s. We observed a decrease in resistance upon initial contact as conductive elements, such as ions, fillers, and polymers, aligned or reorganized at the interface to generate more effective pathways for electron flow; this lowered the resistance temporarily until the material stabilized. After 20 s, P40 achieved a self‐healing efficiency of 97%. Therefore, the newly developed skin‐like electronic material can be used in applications that require immediate electrical self‐healing after extremely damaging events, such as those observed during spaceship launches or in desolated harsh environments, where the damage requires instantaneous repair to bypass potential catastrophic incidents. Figure 1i depicts the relationship between the added fillers and conductivity. Pristine samples exhibit a conductivity of 0.16 ± 0.09 × 10^−3^, which increased sevenfold to 1.1 ± 0.44 × 10^−3^ after PEDOT:PSS incorporation and by 22‐fold to 3.6 ± 0.94 × 10^−3^ after the GO concentration reached 40%. Consequently, we used **P40** in most downstream experimental studies.

Typically, the catechol groups (TA) and reactive hydroxyl groups (GO) coincide well with each other, with adequate adhesion to various interfaces. We investigated the adhesion properties of the skin‐like composites to understand this better (Figure S2d,e, Supporting Information; Figure 1j). We observed that the composites adhered to various surfaces, including polymers, metals, and tissues (Figure S2.d,e, Supporting Information). Adhesiveness was further examined using lap‐shear tests and plotted as a histogram (Figure 1j). These tests indicated that the **P40** variant adhered particularly well to muscle, brass, polyethylene terephthalate (PET), and skin tissue, with adhesion strengths in the range of 20–30 kPa, whereas the adhesion strength was typically below 10 kPa for steel, polydimethylsiloxane (PDMS), heart, and tendon tissues. The superior adhesion observed on the skin was attributed to hydrogen bonds between the OH groups present on keratin and ceramides within the skin tissue and the OH groups on GO and TA.^[^ ^19^ ^]^ Typically, muscle tissue is predominantly composed of glutamine, which comprises two C = O groups capable of creating hydrogen bonds with the multiple OH groups present within the TA structure; in fact, up to 60% of the body's glutamine is stored in muscle tissue.^[^ ^20^ ^]^ PET is also abundant in C = O and OH groups, which might be the reason for its higher adhesion than PDMS. Therefore, **P40** was expected to bind well to these substrates. However, **P40** adhering better to brass than to steel was surprising because the process in both scenarios was typically driven by coordination bonds between the catechol groups present on TA and metals from the metal composites. This was attributed to the more hydrophilic nature of brass compared with that of steel, which in turn resulted in more adhesive oxygen‐negative and hydroxyl groups on the surface. Figure S2f (Supporting Information) demonstrates the stable adhesion of **P40** on dynamic tissue‐like surfaces, including nitrile gloves and porcine skin, even after repeated bending and under wet conditions such as water or PBS. These results highlight the robustness of **P40**’s adhesion mechanism, ensuring reliable performance in challenging dynamic and wet environments.

The adhesion capacity of the composites to muscle tissue combined with the high ionic conductivity of the composites enables their use as conductive tissue fillers to re‐establish both mechanical and electrical properties in damaged electroactive muscle tissue. However, potential graphene leakage and PEO itself may cause tissue irritation, and this has to be tested before moving P40 to the next level in this regard. The impedance of **P40** was extremely close to 64 Ω, which is the impedance of native muscle tissue. Despite the extensive market availability of tissue glues for linking broken tissues, including fibrin, cyanoacrylates, dendrimers, and collagen, none of them are electrically active.^[^ ^21^ ^]^ We capitalized on the electrical benefits of the **P40** variant in healing broken muscle tissue both mechanically and electrically by using it to glue the two separated pieces together (Figure 1k). Interestingly, the **P40** variant successfully re‐established electrical conductivity within the muscles to levels similar to those before damage. This was in stark contrast to the electrical measurements obtained from the samples that were re‐glued using conventional bioglue. The results depicted in Figure 1j,k and Figure S2a–f (Supporting Information) highlight the potential of **P40** as a stretchable, self‐healing, and conductive tissue adhesive for electroactive tissues. Therefore, they can be used to adhere cyborganic implants to native tissues in a gentle manner. The adhesion to a wide range of substrates contributes to the versatility of P40 components in terms of, repairing, maintaining, and replacing broken parts.

### 2.6 Mechanical Analysis

**Figure 2:**
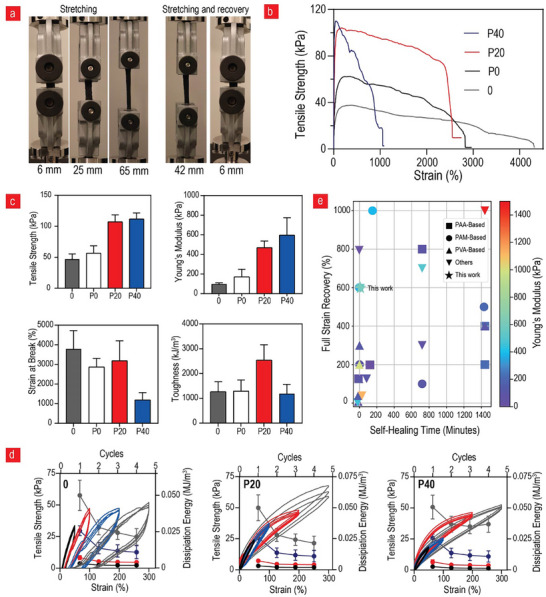
**Mechanical Characterization**. a) Visual representation of the remarkable stretchability of **P40**, exhibiting its ability to extend up to 65 mm from a 6 mm gauge length, with almost complete recovery observed after stretching it to 42 mm. b) Tensile stress–strain curves of the various tested composites. c) Comprehensive mechanical property analysis, including tensile strength, Young's modulus, strain at break, and toughness (n = 4). d) Cyclic mechanical testing and dissipated energy evaluation for varying graphene oxide (GO) filler concentrations (**0**, **P20**, and **P40**) at different strain levels: 50% (black), 75% (red), 150% (blue), and 300% (grey) (n = 4). (e) Relationship between self‐healing time, strain recovery, and Young's modulus with differentiated group markers, comparing the values observed for **P40** with those reported in the literature for hydrogels with both adhesive and self‐healing characteristics.^[^ ^9^, ^24^ ^]^ All data are presented as mean ± SD.

In several cases, materials that can self‐heal swiftly without external aid or materials that demonstrate adhesive properties tend to experience diminished strain recovery and compromised mechanical integrity.^[^ ^7^, ^8^, ^17^ ^]^ This renders it challenging for them to fully harness their potential in real‐world scenarios. To determine whether the proposed material can address this limitation, we performed a series of comprehensive mechanical tests using an Instron mechanical tester in the tensile mode. This facilitated further examination of the efficacy of the developed life‐like electronic materials to explore their potential in overcoming existing methodological gaps in the field. **Figure** **2**a indicates that a partial recovery is observed after extending the **P40** variant to ten times its original length, and an almost complete recovery after extending it to seven times its original length. This is within the same range as other commercially available elastomers in the field, such as styrene ethylene butylene styrene (SEBS), Sylgard 184 PDMS, and polyurethane, which typically maintain full recovery potentials after 200%, 700%, and 280% strain, respectively.^[^ ^22^ ^]^ The mechanical tester provided additional details. As indicated in Figure 2b, the composites can be strained up to ≈4 000% of their original length, with the maximum strain decreasing when more fillers (PEDOT:PSS and GO) are added to the system. Improved mechanical properties were observed after the addition of PEDOT:PSS and GO. For instance, the value of Young's modulus increased almost sixfold from 100 to 600 kPa after the incorporation of 40% GO (Figure 2c). This was expected considering that the Young's modulus of a single graphene sheet is considerably larger than that of soft polymeric matrices. In theory, the formidable strength of graphene can be increasingly exploited with the increase in the concentration inside the composite until a certain limit, which was 40% inclusion in this study. Notably, the obtained Young's modulus values were within the range typically observed for soft tissues, such as skin, skeletal muscle, cornea, and heart tissue, thereby adding to the tissue‐like properties of this new life‐like composite material.^[^ ^23^ ^]^ Conversely, the tensile strength only increased twofold, whereas the toughness values were significantly within the standard deviations (Figure 2c). By contrast, a drastic decrease was observed in the case of strain at failure from pristine material (3 800%) to the **P40** variant (1 000%). Other studies have reported similar observations, and this phenomenon occurs when the spacing between nanomaterials decreases to a critical point, leading to mechanical friction between individual nanomaterial sheets. This can result in mechanical stress propagation and critical material failure at an earlier stage.

Cyclic tensile tests with four cycles up to 600% strain were performed to obtain a more precise understanding of the mechanical recovery properties of the new composites (Figure 2d). In contrast to other rapid self‐healing materials with adhesive properties, we achieved almost complete recovery even after four cycles, with the recovery improving with the increasing GO content. Furthermore, previous studies have reported that complete recovery was achievable only below 100% strain for materials that could self‐heal within minutes, similar to the developed material (Figure 2e; Table S1, Supporting Information). Typically, materials that can recover fully above 100% strain display self‐healing durations of several hours, which is in contrast to the self‐healing time (in minutes) observed in this study (Figure S3b,c, Supporting Information). Most systems that can recover rapidly display subpar stiffness, typically below 200 kPa, which is in striking contrast to the 600 kPa achieved here.^[^ ^9^, ^24^ ^]^ Notably, a near‐complete recovery is only obtained below 100% strain, which is in sharp contrast to the results obtained here. Thus, the developed composite system is novel along these lines.

### 2.7 Physical Sensing

Physical changes such as dimensional modifications, pressure alterations, temperature variations, and pH values are parameters that can be used in the field to adapt to hazards. These changes can also diagnose a wide range of diseases, such as diabetes, circulatory disorders, and breast cancers, as they result in swollen tissues (edema), increased skin temperature, and chronic wounds. To date, detecting such changes has relied on bulky, expensive, easy‐to‐break, rigid, non‐conformable, and unreliable electronics fabricated using magnetometers, gyroscopes, accelerometers, thermometers, pH meters, and bioimpedance spectroscopy. At the core of the existing methodological gap is the lack of methodology in terms of sensors capable of simultaneously monitoring multiple physical parameters. This is because several cues must be simultaneously assessed in both health and robotics applications. In this section, we demonstrate how the **P40** variant can effectively overcome these existing challenges.

**Figure 3:**
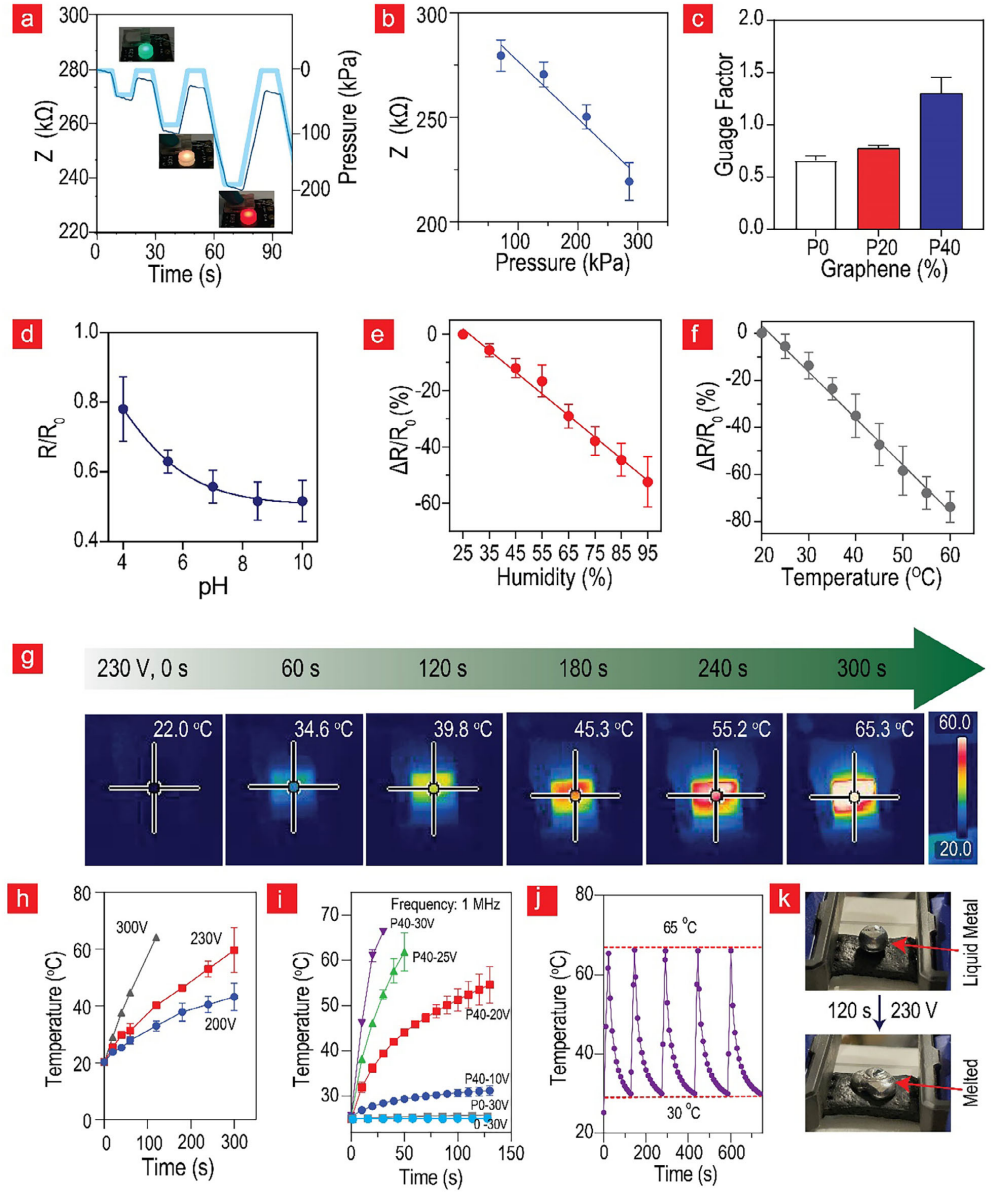
**Physical sensing and heat regulation capacity**. a) Establishment of a stable correlation between pressure and impedance, demonstrating a simultaneous decrease in resistance with the increased pressure. b) Characterization of the relationship between impedance and pressure (n = 4). c) Gauge factor calculations derived from strain measurements of up to 600% strain. The relationships between electrical impedance and d) pH value (n = 4) e), humidity (n = 5), and f) temperature (n = 5), exhibiting the adaptability and sensitivity of **P40** to a broad range of physical parameters. g) Joule heating performance of **P40,** heated from room temperature to 65 °C by applying a DC voltage of 230 V (n = 3). h) Variations in the heating rate as a function of DC voltage (n = 3). i) Joule heating of **0**, **P0**, and **P40** at a resonance frequency of 1 MHz at low voltages (n = 3). j) Cyclic regulation of temperature, exhibiting rapid and reversible temperature changes from 65 to 30 °C (n = 3). k) Effective transformation of liquid metal Gallium into a liquid state within minutes using the observed Joule‐heating property. All data are presented as mean ± SD.

We established a stable correlation between the strain, flexibility versus resistance (Figure S4, Supporting Information), and pressure versus impedance (**Figure** **3**a), wherein the resistance decreased with the pressure and increased during strain and bending. This was expected because the graphene sheets naturally came closer to each other under pressure and moved further apart during straining. Closer proximity extended the characteristic length that a charge could traverse, thereby significantly augmenting the electrical conductivity; the opposite scenario occurred during flexing and straining. The pressure sensing behavior was visualized by coupling **P40** to an LED that could change the light from blue to red as a function of current, with less current resulting in a more bluish light (Figure 3a). This concept can be used in wearables designed for visual monitoring of swollen tissue, as increased swelling due to lymphoedema results in more pressure, thereby switching from a blue LED to a red one. Figure 3b depicts further characterization of the relationship between the impedance and pressure. Here, an almost linear relationship is observed with a sensitivity of 0.15 kPa^−1^, which is comparable with that of various nanocomposites‐based hydrogel pressure sensors (0.01–0.2 kPa^−1^).^[^ ^25^ ^]^ In terms of strain sensitivity, we observed that the gauge factor increased from ≈0.5 to 1.5 as the graphene content increased to 40% (Figure 3c) with stability over 5 000 cycles (Figure S4d,e, Supporting Information).

A similar relationship between the temperature, pH, and humidity was established with respect to the electrical resistance (Figure S5a–c, Supporting Information). These exciting trends were characterized in more detail and plotted (Figure 3d–f). We observed a twofold increase in electrical resistance as the pH value increased from 4.5 to 7.5, a twofold decrease in relative resistance as the humidity increased from 35% to 95%, and an 85% drop in relative resistance as the temperature increased from 20 to 70 °C. Additionally, **P40** displayed excellent sensitivity at − 1.8%/%RH and − 2%/°C toward humidity and temperature, respectively. Their sensitivity range was similar to that of different temperature (− 0.06 to − 6.5%/°C) and humidity (0.1–6%/%RH) sensors composed of soft materials.^[^ ^26^ ^]^ We believe that the pH sensitivity is associated with the protonation of TA at lower pH values, whereas the humidity sensitivity is linked to the increase in the composite water content, which in turn facilitates improved ion transport and conductivity. The temperature sensitivity of the **P40** variant is most likely associated with the incorporation of the highly thermally conductive rGO in the composition.

Additionally, we identified a relationship between pH and color. We observed a gradual transformation from a grayish shade to black as the pH increased from 3.0 to 9.0 (Figure S5d, Supporting Information). This type of correlation can be used on wound bandages to monitor bacterial growth over time because biofilm formation is closely linked to a decline in the surrounding pH value. Notably, a mobile device can be employed to capture such changes over time, as demonstrated in several recent studies in this field.^[^ ^27^ ^]^

### 2.8 Heating

Graphene is a good conductor of both electricity and heat. We capitalized on this property and transformed **P40** into an electrical heater. We achieved this by utilizing the Joule effect, taking advantage of the heat produced by charges accelerated in an electrical field. In other words, the faster the charge carriers move, the more heat they can dissipate after entering the conductor body. Figure 3g illustrates the Joule heating performance of **P40**, which instantly heats from room temperature to 45 °C in 2 min and can reach up to 65 °C by applying electricity. The applied voltage resulted in uniform heat distribution across the **P40** sample, which was most likely because of the even distribution of the highly thermally conductive rGO inside the composite. The heating rate varied with the voltage. As indicated in Figure 3h, less power and heat are generated at a low voltage, in accordance with the working mechanism behind the Joule effect. For instance, when the applied voltage was increased to 300 V, the heating was accelerated because of the increased current transfer through it, reaching 45 °C in 1 min at low power (1 W). The standard EU DC voltage (220–230 V) can also heat the composite to 60 °C in 5 min using only 1.6 W. Such low‐power heat‐producing electrodes can be used in low‐voltage electrocautery (electrosurgery) applications, such as hemostasis, where instantaneous desiccation and coagulation occur when the temperature exceeds 60 °C. Furthermore, they can be used in various health applications, such as low‐level heat therapy, thermal tumor ablation, and in vivo heat treatments. Subsequently, we transitioned from DC to AC voltage and determined the resonance frequency of the circuit to be 1 MHz. The resonance frequency signifies the point at which the inductance and resistance of the circuit intersect, resulting in maximum current flow, which in turn leads to maximum heat generation. Operating at this resonance frequency facilitated the rapid and efficient heating of **P40** at significantly lower voltages. Remarkably, the temperature of **P40** was increased to 70 °C within 1 min using a mere 30 V, and within ≈2 min, a temperature increase of 30 °C was achieved at 10 V (Figure 3i). Such swift heating using soft electronics, such as **P40**, is extraordinary compared with previous reports. In contrast, similar behavior was not observed with **0** and **P0**, indicating that resonance heating is accelerated though the graphene sheets in the composites. Further investigations are required to understand the different frequency responses associated with resonance heating of the composite materials.

Another advantage is the cyclic nature of the observed heating properties (Figure 3i,j). These findings demonstrate a rapid and reversible regulation of temperature in the range of 65–30 °C. This capability presents exciting possibilities for the development of heat‐regulating outer skin surfaces on soft robots or smart suits for astronauts, explorers, and divers. Furthermore, our research shows the effectiveness of the observed Joule heating in transforming liquid‐metal gallium into a liquid state within minutes (Figure 3k). This new digital skin‐like system has the potential to enhance soft robots by enabling them to eliminate obstacles such as ice or other flammable materials encountered in nature or distant destinations.

## 3 3D Printability

We performed a series of rheological measurements to determine whether the proposed material can be subjected to extrusion‐based printing after relevant environmental exposures, such as temperature, acidic environment, and shear rate. Figure S6a–c (Supporting Information) depicts several interesting observations made regarding the behavior of the complex viscosity. We determined that the viscosity of **P40** relied inversely on both pH and temperature, with the viscosity decreasing with an increase in these physical parameters. This property offers the added benefit of on‐demand recycling or degradation via a simple and non‐toxic pathway, in contrast to most electronics available in the market; therefore, **P40** can substantially aid in addressing e‐waste challenges. The temperature stimuli‐responsiveness (Figure S6a, Supporting Information) can be harnessed for controlled 3D printing and drug release. Specifically, pH/temperature stimuli can be used to make an otherwise difficult‐to‐extrude **P40** easier to work with by rendering it more flowable during the printing phase. Moreover, the ability to solidify the printed object through material cooling offers an additional advantage. However, the temperature‐dependent loss of the structural integrity of the polymeric matrix can be used to facilitate drug release as molecules encounter reduced resistance from the surrounding material.

Our findings revealed that the composites exhibited shear‐thinning behavior at 100/s, a value within the range typically required for extruding liquids from 28 Gauge needles (Figure S6c, Supporting Information). As explained in Figure S6d–f (Supporting Information), we utilized these unique rheological properties to print some complicated structures via both extrusion printing and screen printing. Figure S7 (Supporting Information) depicts the freeze‐dried **P40** used to print delicate electronics and motion sensors. A drawback of 3D extrusion printing is the inherent difficulty of printing multilayer structures. These challenges primarily originate from two factors: the material spillover that occurs after the printing phase and the structural limitations of large printed objects. These limitations can be attributed to the generally low Young's moduli of soft and extrudable materials, which hinder their ability to support their own weight. Overcoming these limitations is crucial for enhancing the feasibility of extrusion printing complex and sturdy multilayer structures.

**Figure 4:**
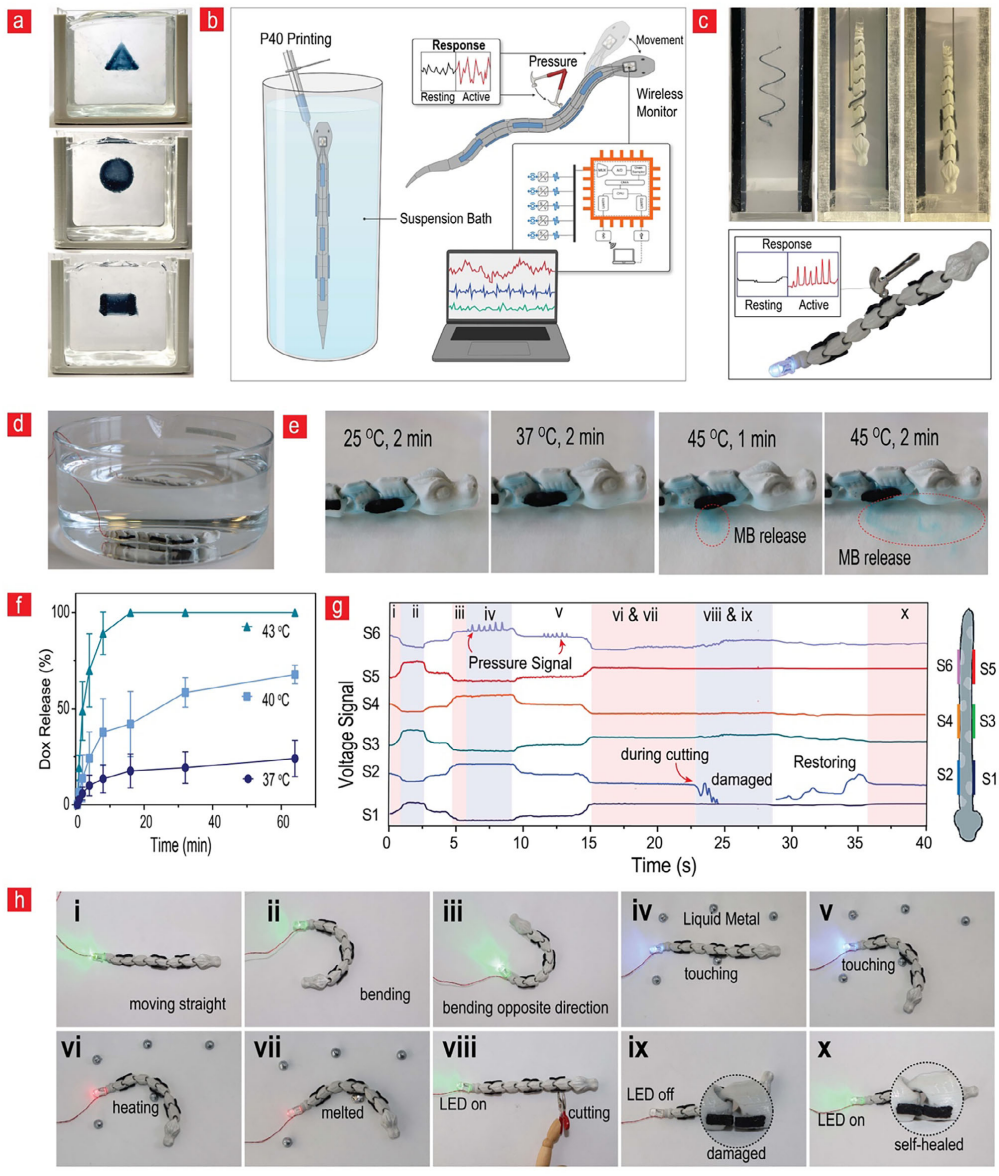
**Exploration, military, and medical robotic applications**. a) Suspension printing of complex structures, including a pyramid, sphere, and square. b) Use of a support bath for 3D printing of sensors on a robot. c) 3D printing the sensors onto a complex snake structure using a laponite‐based medium as support. A representative image showing that the **P40**‐equipped snake can retransmit pressure data originating from hammer hitting. d,e) Visual presentation of the drug‐releasing properties of **P40** by evaluating its potential as a temperature‐triggered system for the release of methylene blue. f) Assessment of the temperature‐dependent release behavior of doxorubicin to probe the drug delivery potential of **P40 (**n = 4). g, h) Connecting the measured voltage changes (n = 3) with representative images of various scenarios showing the role of **P40** as an artificial digital skin for an exploratory snake with pressure‐ and motion‐sensing capabilities and obstacle‐avoidance capacity via heating. All data are presented as mean ± SD.

One promising solution for overcoming these challenges is the use of 3D printing in suspension baths. In this approach, printing occurs within a semi‐solid medium, which eliminates the material spillover issues often encountered when printing with thin air as the surrounding medium. Therefore, this method offers more precise control over the extrusion process, ensuring improved printing quality and enabling the printing of larger structures. We optimized this approach (Figure S6g,h, Supporting Information) to print complex structures, including a pyramid, sphere, and square. In particular, realizing sphere‐like architectures via conventional 3D printing on 2D substrates is difficult owing to the structural collapse caused by the weight of the overhanging layers (**Figure** **4**a). However, we capitalized on this property to print pressure and motion sensors onto an artificial snake (Figure 4b,c). We successfully printed complex curvilinear spirals and interspaced rectangular structures in 3D using the interface between the snake and support bath (Figure 4c). The 3D‐printed **P40** material exhibited excellent adherence to the snake body, demonstrating its inherent capacity to adhere to polymeric surfaces (Figure 1j). After printing, the snake was removed from the support bath and connected with wires and a data transfer module to transmit pressure and motion data to a portable device. The **P40**‐equipped snake robot was functional and provided a high‐fidelity readout after being repeatedly hit by a small hammer (Figure 4c). This feature can also be used in field applications for sensing important encounters with obstacles, which can enhance the performance of exploratory robots by enabling them to rapidly adapt to new situations.

### 3.1 Robotics Applications

The aforementioned capabilities of **P40** confirm its promising future as a potential self‐maintainable and multi‐responsive artificial electronic skin interface that can detect and respond to multiple stimuli, thereby opening up a range of applications in the fields of medical and soft robotics. Figure 4 illustrates some of these unique applications. Figure 4d,e illustrates the drug‐releasing properties of **P40**, providing valuable insights into its potential as a Joule‐heating‐based drug delivery or toxin‐releasing system for medical and military applications. The incorporation of methylene blue served as an initial probe to evaluate the response of **P40** to varying temperatures. At physiological temperatures of 25 and 37 °C, we observed that the **P40** variant demonstrated a notable resistance to releasing methylene blue into the surrounding medium. These findings suggest that the **P40** variant maintains its structural integrity and drug encapsulation properties under normal physiological conditions, which are crucial for an effective drug delivery system. However, the significant release of methylene blue observed at 45 °C indicates a sharp transition in its behavior in response to elevated temperatures, indicating that electrical stimuli and Joule heating can be used to release the drug on demand. This characteristic can be leveraged to achieve targeted drug release in regions of the body at elevated temperatures, such as in hyperthermia‐based cancer therapies or in areas undergoing inflammation. Furthermore, the successful incorporation and release of doxorubicin, a potent chemotherapeutic agent, into **P40** at various temperatures further underscores its potential as a versatile drug delivery carrier. The rapid and complete release of doxorubicin within 2 min at 45 °C is particularly noteworthy (Figure 4f). This signifies that it can potentially serve as a platform for achieving highly efficient and controlled delivery of therapeutic or chemical agents, particularly in situations where precise and rapid release is essential. Moreover, by harnessing the potential of Joule heating, this new class of material can be employed in conjunction with endoscopes, enabling precise drug delivery to specific anatomical sites while burning away unwanted tissue or tumors.

Figure 4g,h depicts the **P40** variant used as a self‐maintainable electronic skin on a robotic snake to demonstrate its capabilities in terms of sensing pressure and detecting changes in motion and responding to them. We introduced this robotic snake into an obstacle course filled with liquid metal balls and assessed its capacity to sense, adapt, and respond to obstacles through a self‐maintainable artificial skin covering. We observed specific changes in the voltage from the snake sensors (Figure 4g), corresponding to the specific scenarios depicted in Figure 4h (the actual robotic snake used for the data in Figure 4g is depicted in Figure S8, Supporting Information). Figure 4h presents a series of ten images corresponding to the scenarios depicted in Figure 4g. The images show a snake moving between obstacles and transmitting different motion patterns via the associated voltage change, as depicted in Figure 4h for scenarios (i)–(iii). A total of six sensors were attached to the robotic snake, as shown in Figure 4g, with both ends of each sensor connected to tin electrodes, which were then interfaced with a PCB board for capturing voltage signals during different events. When bending occurs, the resistance changes depending on the direction of movement, which is reflected in the voltage signals. During rightward movement (Scenario ii), sensors S2, S4, and S6 experience compression due to bending‐induced shrinkage, leading to a decrease in resistance and a corresponding drop in voltage signal, whereas sensors S1, S3, and S5 undergo strain due to bending‐induced stretching, resulting in an increase in resistance and a rise in voltage signal. Conversely, during leftward movement (Scenario iii), the opposite trend is observed. This variation in ADC values enables clear differentiation between rightward and leftward bending motions. In scenarios (iv) and (v), we programed an LED lamp to detect liquid‐metal‐based obstacles by enabling the lamp to change the light from green to blue in response to the pressure changes experienced by the sensors in a similar vein, as illustrated in Figure 3a. When the sensor comes into contact with an obstacle or experiences forces, the material undergoes bending, which disrupts the conductive pathways and leads to an increase in resistance. This resistance change results in a corresponding rise in voltage signals. The magnitude and nature of the voltage increase depending on whether the event is an impact or sustained force. Hammer‐like impacts induce high‐amplitude, transient voltage spikes due to the rapid and forceful deformation of the material, whereas continuous force leads to a gradual, steady increase in voltage as the material remains bended. This distinction enables differentiation between dynamic impact and sustained events. Furthermore, by analyzing sensor responses, it is possible to distinguish localized effects, as only the sensor in direct contact with the obstacle exhibits a voltage increase, while others remain unaffected. To obtain clear visibility of the pressure signal, the pulse signals presented in Figure 4g (scenarios (iv) and (v)) were generated by repeated hits with a hammer at different pressure levels. To demonstrate the adaptive nature of the sensor, we investigated its response to mechanical pressure followed by a thermal stimulus. After sensing the obstacles, the P40 sensor was subjected to Joule heating in scenario (vi), resulting in a current‐induced temperature rise, which triggered a programed LED color change from blue to red. This heating mechanism, based on the Joule heating principle is described in Figure 3, played a crucial role in melting the liquid metal obstacles during the experimental scenario (vii). To achieve this, we applied an AC voltage at 1 MHz upon contact with obstacles to induce localized heating. Initially, the robotic snake detects the obstacle through tactile feedback and subsequently applies voltage to initiate the melting process. This sequential operation allows for a clear distinction between thermal events and mechanical deformations, as heating does not produce immediate changes in voltage signals in the same manner as bending or force‐induced effects. The remarkable aspect of this material was its inherent self‐healing properties, which were observed in scenarios (viii), (ix), and (x), and the associated voltage signals in Figure 4g. As observed in Figure 4h (scenarios (viii), (ix), and (x)) and Figure 4g, the self‐healed P40 variant recovers its voltage signal within 20 s. This process can be repeated several times, as illustrated in Figure 1h. This unique characteristic significantly enhances the potential of the material for use in the development of next‐generation digital machinery with self‐maintenance capabilities. The incorporation of skin‐like electronics into exploratory soft robotics exhibits significant potential for advancing the field by enabling self‐maintenance beyond mere self‐healing through the capability of sensing, responding, and adapting to the environment.

Figure S9 (Supporting Information) illustrates the potential of these life‐like material systems for defense applications, wherein a command received from a satellite triggers the release of toxins by heating the material via Joule heating. The second part describes the pressure sensor and Joule heating properties of **P40**, which are employed by exploration robots working in underground tunnels to detect and melt obstacles such as ice. Figure S9 (Supporting Information) further depicts another possible application associated with the pH sensitivity and drug release capabilities of **P40**‐coated snake‐like microrobots. For instance, these microrobots can move inside the body to detect tumors while monitoring their microenvironments with the inherent capacity to release drugs on demand to kill cancer cells. Here, the pH value of the microenvironment is a crucial marker for the well‐being of the tumor and can readily be probed by **P40**. It can then be used to estimate when and what quantity of the payload should be released.

### 3.2 Biomolecule Sensing

**Figure 5:**
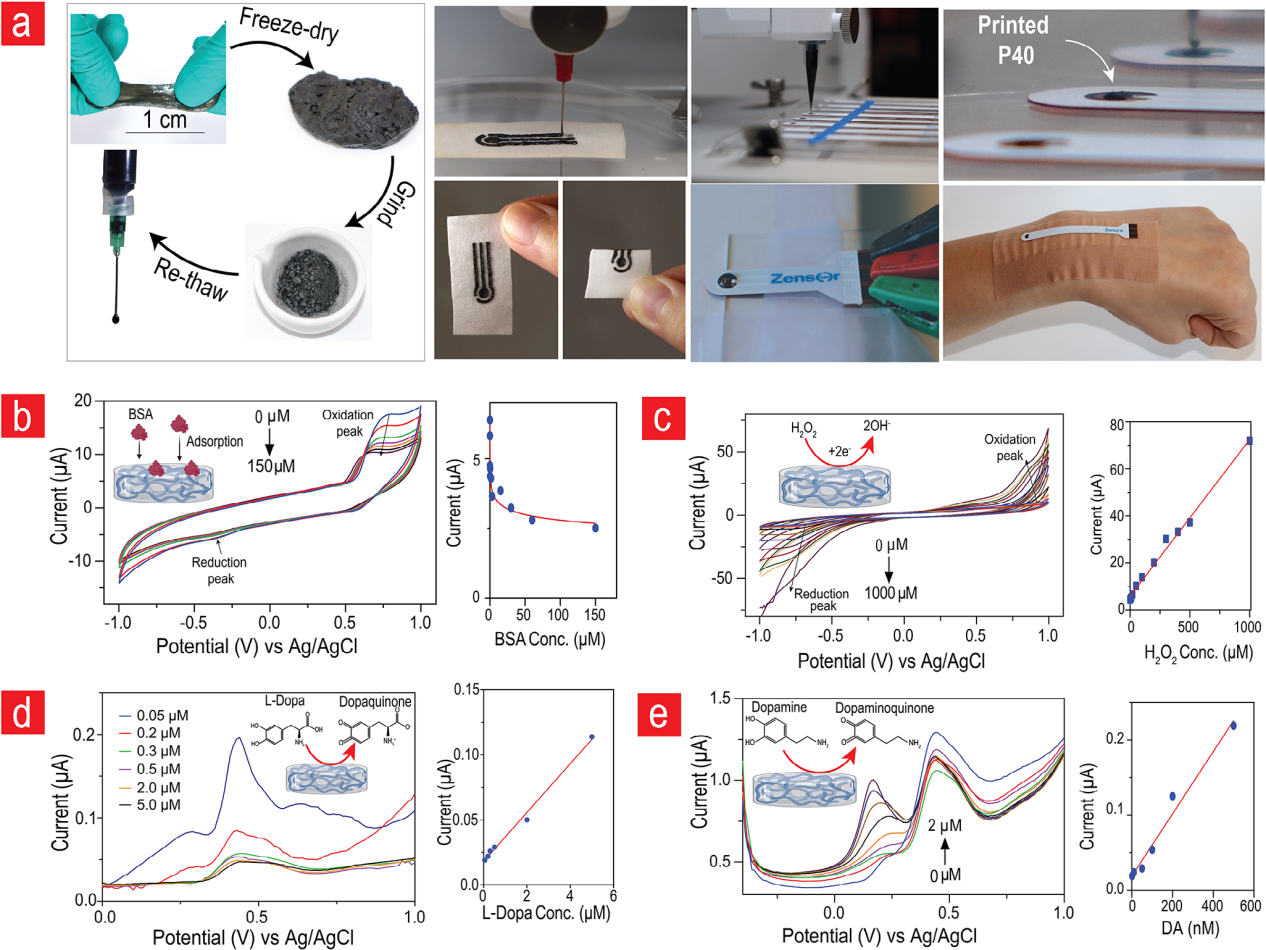
**Advanced biosensing applications of P40**. a) A highly scalable 3D extrusion printing method for integrating **P40** into wearable electrochemical‐based biosensors. The use of cyclic and DPV on **P40** for sensing b) BSA, c) hydrogen peroxide (H_2_O_2_), d) L‐dopa, and e) dopamine. All data are presented as mean, n = 4.

The unique multi‐adherence and shear‐thinning properties of **P40** render it easily customizable to different wearable interfaces via 3D printing. This presents a cost‐efficient avenue for maintenance using 3D‐printed broken parts and their easy integration into wearables owing to the unique adherence and moldable properties of **P40**. To tap into this potential, we used the 3D printing method presented in Figures S6 and S7 (Supporting Information), specifically the extrusion printing of freeze‐dried and re‐thawed **P40** (**Figure** **5**a). Initially, we demonstrate the ease of printing **P40** onto a bandage and its subsequent stable adherence to the bandage interface. Furthermore, we successfully printed the **P40** material onto a commercially available flexible electrochemical three‐electrode sensor from Zensor, ensuring seamless integration with the sensor. The resulting setup enabled precise electrochemical measurements of various biological markers in addition to the physical markers shown in Figure 3.

We applied cyclic voltammetry (CV) and differential pulse voltammetry (DPV) on **P40** and observed the unique redox behavior associated with Graphene:PEDOT:PSS corresponding to oxidation and reduction peaks at 0.6 V and − 0.4 V, respectively (Figure 5b). This behavior established the basis for subsequent biomolecule‐sensing experiments. BSA molecules were selected as the initial target for analysis. In numerous clinical and biomedical applications, the detection of serum albumin is of the utmost importance because accurate quantification is essential for the diagnosis and monitoring of a wide range of diseases and physiological conditions.^[^ ^28^ ^]^ A significant change was observed in the CV response after the addition of BSA. Figure 5b indicates that the intensity of the redox peak decreases with the increase in BSA concentration. This trend suggests that the BSA molecules adsorb onto the surface of the electrode and alter the electrochemical behavior of **P40**. Furthermore, we used **P40** for hydrogen peroxide sensing; hydrogen peroxide is an important marker during acute inflammation and myocardial infarction as well as in some tumor microenvironments.^[^ ^29^ ^]^ The results revealed that the reduction current associated with hydrogen peroxide detection occurred at ≈ − 0.8 V when 1 µm of H_2_O_2_ was introduced. This finding confirms that the **P40** variant demonstrates effective hydrogen peroxide detection capabilities at this specific voltage. As the concentration of hydrogen peroxide gradually increased, a corresponding increase in the reduction current was observed (Figure 5c). This trend indicated a concentration‐dependent response in which higher concentrations of H_2_O_2_ led to an amplified electrical current associated with the reduction process. Subsequently, we used DPV to evaluate the ability of **P40** to quantify the concentrations of L‐dopa and dopamine (Figure 5d,e). Both L‐dopa and dopamine are important biomarkers of various neurodegenerative diseases. To demonstrate the performance of the flexible electrodes, L‐Dopa detection was carried out using commercial Zensor flexible screen‐printed electrodes, while all other studies were performed on **P40**‐attached glassy carbon electrodes in a three‐electrode setup. The electrochemical response exhibited a distinct peak at ≈0.6 V, indicative of the PEDOT response to these molecules. Upon the addition of L‐dopa to the solution, a noticeable increase in the peak response was observed, which was characterized by a distinct peak at ≈0.55 V. This significant shift in the peak potential suggests a specific and selective interaction between L‐dopa and the **P40** matrix, which in turn modified the electrochemical behavior. Notably, the response exhibited excellent linearity up to concentrations of 5 µm and sensitivity down to 50 nm, demonstrating the potential for the precise and quantitative detection of L‐dopa within this concentration range. Conversely, dopamine exhibited a distinct peak at ≈0.2 V, with the intensity increasing proportionally with increasing dopamine concentrations. At concentrations up to 500 nm, the response exhibited a good linear relationship, thereby establishing a reliable dopamine detection range. Furthermore, CV measurements were performed to evaluate the sensitivity of the ascorbic acid detection (Figure S10, Supporting Information). Ascorbic acid induced an increase in the response at 0.6 V, further demonstrating the sensitivity of the **P40** material to ascorbic acid within the concentration range examined. This response exhibited a linear relationship with the ascorbic acid concentration up to 50 µm, indicating the potential for accurate quantification within this range.

The **P40** variant demonstrates immense potential as a biomolecular sensor, particularly in the field of healthcare and diagnostics. The flexible 3D‐printed **P40**‐based electrochemical setup (Figure 5a) can seamlessly interface with micro‐needle setups, facilitating closed health‐maintaining loops. In the future, **P40** can be used for the real‐time sampling of crucial health‐related biomarkers from subcutaneous fluid reservoirs with the capacity to respond to the biomarkers by releasing drugs on demand via electrical heating, as illustrated in Figure 4d–f. Similarly, by integrating **P40** into hygiene pads or underwear, fluid samples can be efficiently collected and analyzed, enabling health monitoring through biochemical composition analysis. Moreover, when integrated into socks or other textiles, it can monitor dimensional changes associated with edema or temperature fluctuations, which are indicative of circulatory issues such as deep vein thrombosis. The scalability and easy disassembly of **P40** materials via temperature and pH regulation (Figure S6a,b, Supporting Information) can potentially revolutionize healthcare by enabling the development of sustainable and cost‐effective healthcare products. This aligns with the circular economy principles of the future, in which universal access to readily available healthcare products is combined with environmentally conscious practices. In summary, **P40**‐based digital wearables can not only improve healthcare, but also ensure that it is more accessible, affordable, and in harmony with a sustainable future.

## 4 Discussion

In this study, we defined the term self‐maintainable material system, and explored its potential applications in various fields. This system can autonomously preserve and maintain themselves in diverse environments via a wide range of functionalities, including high mechanical performance, durability, and self‐healing properties, combined with the ability to sense, respond, and rapidly adapt to environmental changes. Our analysis confirmed that **P40** satisfies the aforementioned requirements, can be printed and molded into almost any shape, and can adhere to both organic and inorganic interfaces. This adds to the list of **P40** properties that are conducive to maintenance. For instance, engineers can use 3D printing tools and customize devices according to specific requirements, thereby enhancing their longevity and functionality. Moreover, the ability to swiftly fabricate malfunctioning **P40** components on‐site coupled with their strong adherence characteristics simplifies the repair and reintegration of broken parts into malfunctioning devices.

As a proof‐of‐concept, we printed **P40** as digital skin on robots and demonstrated that this can endow them with self‐maintenance capacity by allowing them to heal, sense, respond, and adapt to challenging environments, similar to the behavior of living organisms (Figure 4). We further demonstrated that **P40** can pave the way for new avenues in medicine by enabling the on‐site manufacturing of wearable healthcare sensors with the potential to enable prints that can readily blend in with patient‐specific anatomical features, thereby facilitating a better recording of health‐related parameters for diagnostic purposes.

Fundamentally, we demonstrated that **P40** goes beyond the current state‐of‐the‐art by enabling rapid and autonomous self‐healing within minutes despite its high elasticity (full strain recovery after 600% elongation). Although significant advancements have been reported in self‐healing materials such as semi‐conductive polymers, conductive polymers, and liquid metal‐based systems, most of them suffer from limitations, including long healing times, reliance on external stimuli (e.g., heat, light, or electricity) for repair, and excessive softness and fragility.^[^ ^30^ ^]^ However, **P40** can overcome several of these challenges, contributing to the development of adaptable and versatile electronics. By addressing these limitations, **P40** can significantly enhance the longevity of machines, robots, and consumer electronics.

Additionally, **P40** exhibits adherence properties and can reconnect broken tissues. Although non‐toxic tissue glues are available in clinical practice, they lack the necessary mechanical toughness and electrical properties required for healing electroactive tissues, such as muscle, cardiac, and nerve tissues. In this regard, **P40** serves as a unique material capable of mending both the mechanical and electrical aspects of the damaged electroactive tissue. Importantly, the strain‐gauge properties of **P40** render it ideal for monitoring the dynamics of healed tissues during movement. Moreover, they offer exciting potential for controlled drug release, presenting new avenues for theragnostic applications where regenerative drugs can be released during the healing process or when tissue movement is suboptimal.

Apart from its combination of adhesion capacity, printability, and self‐healing properties, the uniqueness of **P40** lies in its remarkable electronic properties, which enable it to regulate heat through reversible and cyclic Joule heating. Additionally, the inherent electronic properties of **P40** have enabled it to sense a wide range of chemical, physical, and biological markers. Although several Joule heaters are available that utilize highly conductive metals and nanocarbon materials to achieve high performance at lower DC voltage levels (< 15 V), the effectiveness of Joule heating on ionic conductive materials such as **P40** is typically limited, often requiring the use of AC currents at significantly higher voltages. For instance, a recent study by Zhang et al. (2022) reported a flexible ionogel‐based Joule heater that reached temperatures up to 180 °C using a considerably higher AC voltage (150 V) in a longer time period of 80 s.^[^ ^31^ ^]^ Another study by Shi et al. (2023) demonstrated Joule heating of a simple ionic salt solution to 70 °C using a higher AC voltage (118 V) at 10 kHz.^[^ ^32^ ^]^ Considering the values reported in these studies and others in the literature, the developed **P40** material is among the best ionic Joule heaters available.^[^ ^31^, ^32^, ^33^ ^]^ These excellent Joule‐heating properties can be attributed to running the AC voltage across **P40** at its electrical resonance frequency, where the capacitance and inductance are equal, resulting in maximum current flow and therefore ensuring maximum Joule heating.^[^ ^34^ ^]^ This resonance frequency correlates with the vibrational states of a few hydrogen bonds present in the material, leading to increased near‐infrared emittance and enhanced heating.^[^ ^35^ ^]^ Consequently, this vibrational heating can promote a more mobile phase in the polymeric matrix, thereby reducing the resistance to the mobility of conductive ions within the matrix. This combined effect explains the efficient heating capabilities of **P40**.

In terms of physical sensing, **P40** demonstrated sensitivity on par with other temperature, pH, and humidity sensors available in the market. In terms of biochemical sensing, **P40** delivered impressive results in the µm range for BSA, hydrogen peroxide, L‐dopa, and ascorbic acid, with dopamine sensitivity in the nanomolar range. Several sensors based on carbon nanotubes, graphene, metal organic frameworks, metal oxides, Au nanoparticles, and Ag nanoparticles are available, which offer higher sensitivities in the nm and pm range; however, the sensitivity of **P40** is sufficient for detecting these components in human fluids,^[^ ^36^, ^37^ ^]^ where the quantities of the aforementioned molecules typically fall in the range of 0.8–6 µm (H_2_O_2_), 6 nm–60 µm (L‐dopa), 150–770 µm (albumin), 40–200 µm (ascorbic acid), and 0.3–5 nm (dopamine).^[^ ^36^, ^38^ ^]^ As **P40** is a multiplex sensor, a trade‐off may exist in terms of sensitivity. However, its versatility is highlighted by the fact that **P40** is 3D printable and can be easily incorporated into various electronic settings. We successfully integrated these sensing capabilities into flexible electrochemical devices and conventional bandages. Importantly, we have conducted stability tests demonstrating that our encapsulated sensors maintain functionality for up to 7 days (Figure S11, Supporting Information). While further testing is required to confirm long‐term stability, existing literature indicates that employing advanced encapsulation techniques can significantly enhance sensor longevity. For instance, encapsulating hydrogel‐based sensors with materials like Ecoflex has been shown to preserve functionality for over 30 days.^[^ ^39^ ^]^ Additionally, the development of thin‐film encapsulation methods has emerged as an effective strategy to produce reliable coatings that meet the durability needs of flexible electronics.^[^ ^40^ ^]^ These findings suggest that with appropriate encapsulation strategies, our sensors could achieve even longer operational lifespans. Moreover, the high adaptability of **P40** opens up possibilities for self‐maintainable digital patches, as they are flexible and difficult to break, identical to human skin. Furthermore, they are capable of monitoring a wide range of healthcare parameters and responding to them to maintain the well‐being of the wearer, similar to natural skin. Finally, the scalability, cost‐effectiveness, and recyclability of **P40** can facilitate the mass production of wearable devices for healthcare monitoring without affecting the environment through the generation of toxic electronic waste.

## 5 Conclusion

In this study, we present an economical, green, and mass‐producible approach for next‐generation adaptable and versatile soft electronics. The study findings indicate that the production price of **P40** is less than 140 USD/kg for up to 3 000 sensors, with a production time of less than an hour. This affordability renders **P40** an attractive option for various commercial applications, setting it apart from its more expensive and complex rivals such as PEDOT:PSS (≈2 000 USD/kg), polyaniline (≈10 000 USD/kg), poly(N‐isopropylacrylamide) (≈80 000 USD/kg), and polypyrrole (≈15 000 USD/kg). This scalability can be achieved using high‐throughput techniques, such as roll‐to‐roll processing, molding, and customized 3D printing, which support rapid, large‐scale production with minimal waste. This combination of scalability and low cost can contribute to evolving Industry 4.0 to new heights, enabling mass production of electronics with a broad range of applications in human–machine interfacing, flexible electronics, soft robotics, and cyborganics. A key aspect of introducing this material into these fields is its high degree of customization, facilitated by its ability to be easily printed onto intricate 3D architectures and its moldability akin to Play‐Doh. This adaptability extends the usability of the material to remote locations, such as Antarctica, International Space Stations, the Moon, and Mars.

Most existing soft electronic systems are prone to tearing, puncturing, and other forms of mechanical failure, which significantly hinder their use in space, military, and autonomous field robotics applications. **P40** addresses this critical limitation in the field of soft electronic materials. Although rigid shields and packaging methods can enhance the durability of materials, they compromise the inherent softness and flexibility of the systems. However, **P40** introduces a breakthrough soft matter system that possesses immediate self‐repair capabilities even under extreme damage. This unique ability not only addresses the limitations of existing electronic materials, but also opens new possibilities for creating life‐like electronic devices. In addition to its self‐repair capabilities, **P40** has an impressive range of material properties. It exhibits multiplex responsiveness and record‐breaking heat regulation properties. This unique combination of properties can potentially advance civilization in space. These materials are self‐maintainable because they possess sensory capabilities, responsive capacities, and adaptability similar to those of biological systems. In the future, we envision colonies on the Moon and Mars utilizing these life‐like and highly scalable electronic materials rather than conventional wood or metals.

## 6 Experimental Section

### Preparation of rGO

GO powder was prepared according to the method described in the previous work.^[^ ^7^ ^]^ rGO was prepared by the chemical reduction method using TA. In particular, 300 mg of freeze‐dried GO was dispersed in 50 mL of deionized water and subsequently sonicated for 6 h to form a homogeneous brown solution. Then, the GO solution was mixed with 400 mg of TA dissolved in 50 mL of deionized water and was continuously stirred at 85 °C for 9 h. The obtained solid was washed by 40 mL of deionized water and freeze‐dried for 12 h to obtain rGO.

### Preparation of Conductive Poly(ethylene oxide) (PEO) and TA Composites

First, a 2% polyethylene glycol 1 000 000 (SERVA) solution was prepared by dissolving the powder in deionized water and keeping it in a refrigerator for 48 h. Second, stock solutions of 20% TA (Sigma‐Aldrich) and rGO at a concentration of 5 mg mL⁻^1^ were prepared by dissolving TA powder in water and dispersing rGO in water, respectively. Then, the solutions were sonicated for 90 min. PEDOT: PSS solution (1.3 wt.%) was purchased from Sigma‐Aldrich. Samples were prepared using the above stock solutions. E.g., for **P40** samples, 15 mL of PEO solution was stirred with 1.5 mL of PEDOT: PSS for 1 h, then 24 mL of rGO solution was added and stirred further for 4 h. The rGO to PEO powder ratio was kept at 0, 20, and 40 wt.% for different rGO compositions. Then 7 mL of TA solution was added and mixed with a spatula. Then, the precipitate material was collected as gently as possible; it was immersed in water until the unreacted material was removed, and extra water was squeezed and air‐dried. The prepared samples were named **0** (PEO‐TA without PEDOT:PSS and rGO), **P0** (PEO‐TA‐PEDOT:PSS without rGO), **P20** (PEO‐TA‐PEDOT:PSS with 20% rGO), and **P40** (PEO‐TA‐PEDOT:PSS with 40% rGO).

### Chemical Characterizations

The water content was determined by the gravimetric method based on the mass loss before and after freeze‐drying. The weights of the samples were recorded before (W_0_) and after (W_d_) the drying process using an electronic balance. The water content (%) was calculated using the formula:

(1) $$\mathrm{Water}\;\mathrm{content}\;\;\left(\%\right)=\frac{W_{0}-W_{d}\;}{W_{0}}\;\times 100$$

The Fourier transform infrared (FTIR) spectroscopic analysis was by a PerkinElmer Spectrum 100 FTIR spectrometer (USA) with a diamond crystal attenuated total reflectance (ATR). Transmittance mode data was acquired at a resolution of 4 cm^−1^ with 16 scans between 4 000–600 cm^−1^. The x‐ray diffraction (XRD) analyses were carried out in a Huber G670 powder diffractometer (Germany) at 3 to 80° in 2*θ* step‐scan mode with a step size of 0.005° (CuKα1 radiation, λ = 1.54056 Å, operated at 40 kV and 40 mA). Data was acquired in the transmission mode from a rotating flat plate sample oriented 45° to the primary beam. rGO powder and the samples were prepared and mounted onto an amorphous tape for sample analysis. Differential scanning calorimetric (DSC) analysis was performed in order to investigate the thermodynamic property in terms of transition temperature and the heat flow associated with all the phase transitions, melting, and crystallization processes. For this analysis, a DSC Q200 TA Instrument (USA) was employed, where 3–4 mg of samples hermetically sealed in Tzero aluminum pans were weighed and empty pan was used as a reference. The test parameters included a scanning range from − 40 to 250 °C with a scanning rate of 10 °C min⁻^1^ and a constant atmosphere of nitrogen at 50 mL min^−1^. Thermogravimetric analysis (TGA) was carried out to investigate the thermal stability and degradation behavior of the freeze‐dried samples by monitoring changes in sample weight as a function of temperature. The analytical testing was conducted using a TGA Q500 TA Instrument (USA) within a temperature range of 30–800 °C and a scan rate of 10 °C min⁻^1^ along with a constant flow of 60 mL min^−1^ of nitrogen. Scanning electron microscopic (SEM) images were obtained using AFEG 250 Analytical ESEM (FEI Quanta FEG 250) (USA) equipped with field emission gun electron source. All samples were freeze‐dried and sputter coated with gold (10 nm thickness) prior to imaging.

### Mechanical and Rheological Characterization

Tensile, cyclic tensile, lap‐shear, and self‐healing tests were performed using an Instron 5967 Universal Testing System equipped with BlueHill V3 software (UK). The tensile tests were conducted on 14 × 5 × 1 mm (length × width × thickness) specimens, keeping the gauge length as 6 mm. The specimens were elongated at 100 mm min⁻^1^ speed using a 500 N load cell until failure. The stress‐strain curve was generated using Bluehill 3 testing software, which facilitated the determination of tensile strength, Young's modulus, and strain at break. Young's modulus was derived from the slope of the linear region of the stress‐strain curve within the strain range of 0.0%–0.4%, and the toughness was quantified using OriginPro V9.6 software. Tensile cyclic testing was also carried out on 14 × 5 × 1 mm samples with a 6 mm gauge length at strain amplitudes of 50%, 100%, 200%, and 300% at a speed of 50 mm min⁻^1^, and energy dissipation was quantified using OriginPro V9.6 software. The mechanical self‐healing evaluation was carried out to establish the healing effectiveness, which was quantified by the analysis of elongation of specimens. Two sets of composite samples (**0** and **P40**) were prepared by cutting them into 14 × 9 × 1 mm dimensions, followed by gently attaching them to each other for a period of 2 min. The final dimensions of the specimen were 28 × 9 × 1 mm, which was mounted in the machine using a gauge length of 3 mm, and the tensile testing was carried out at a speed of 50 mm min⁻^1^.

The adhesion property of **P40** was evaluated on various substrates according to the ASTM F2255 standard lap‐shear testing method. The substrates tested included steel, brass, PET, PDMS, skin, muscle, heart tissue, and tendon. The samples were 25.4 × 12.7 mm, and the setup was placed under 100 g weight in 37 °C PBS for 10 min. Samples were subjected to tensile testing at a rate of 50 mm min⁻^1^ using an Instron mechanical tester with a load cell of 500 N.

The viscoelastic characteristics of the composites were examined using a Discovery HR‐2 TA Instruments rheometer with parallel plates 20 mm in diameter and with a gap size of 1 mm, controlled at a temperature of 25 °C. The linear viscoelastic region (LVE) was determined through the use of an amplitude sweep from 0.1% to 100% at a constant angular frequency of 10 rad s⁻^1^ and then an angular frequency sweep test between 0.01 and 150 rad s⁻^1^ at a constant strain of 0.1%.

The self‐healing of the composites was characterized by assessing the storage modulus at low strain as a function of its initial value. In this context, the strain step tests were carried out at frequencies of 0.1% to 200% strain (angular frequency: 10 rad s⁻^1^) for each step with the intervals between the steps for 120 s. To analyze the rheological behavior of **P40** in different pH conditions, the samples were immersed in buffer solutions with pH 4, 7, and 10 for 5 min before testing. Dynamic thermal analysis was performed at a constant angular frequency (50 rad s⁻^1^) and 1% strain using temperature ramps in both upward and downward directions from 20 to 60 °C. The shear rate was applied at 25 °C within the range of 0.1–100 s^−1^ to determine the complex viscosity.

The self‐healing behavior of **P40** was visualized by SEM to study the morphological properties of the healed zone in the **P40**. A sharp blade was used to cut the **P40** into two separate parts, which were then attached together. After healing, the sample was frozen in liquid nitrogen and then freeze‐dried. The sample was coated with 10 nm of gold using the Quorum Coater Q150T, in preparation for SEM analysis using the AFEG 250 Analytical ESEM (FEI Quanta FEG 250) (USA).

### Additive Manufacturing

Molding process: The **P40** material was shaped by using a laser‐cut PMMA sheet. The laser‐cut PMMA sheet was used as a pattern, and the **P40** was pushed into the cut pattern to form the desired shape. The shaping process allowed for the creation of precise and intricate shapes for the **P40**.

Screen‐printing: Screen‐printing was utilized to apply the **P40** onto a fabric. The screen mesh was created by laser cutting a PMMA sheet, which was then placed on top of the fabric. To apply the material, a **P40** solution was made with high‐pH (by adding NaOH to the precursor until it reach to pH8) onto the screen mesh, and a squeegee was used to fill the pattern. After the pattern was filled, the pH was decreased by pouring 0.1 m HCl solution. This process allowed to accurately and efficiently apply the **P40** onto the fabric, creating the desired pattern.

3D Printing: The **P40** composite was loaded into a metal cartridge. Using the Hyrel EngineHR 3D printer and applying pressure to the cartridge via a plunger, the material was forced through the printer's nozzle. The material was extruded at a temperature of 50 °C, which allowed for consistent printing.

The **P40** material was freeze‐dried and ground into a powder using a mortar. It was then mixed with a Lutral solution at 4 °C. The Cellink BioX 3D printer was utilized for printing, which involved the applying of 20 kPa pressure via compressed air to accurately and efficiently bind the **P40** powder and Lutral solution. The printed pattern was immersed in cold water to dissolve the Lutral solution. This process enabled the creation of complex **P40** structures.

A 3% laponite solution was mixed and centrifuged to remove air bubbles and used as a printing bath. **P40** material with high pH, achieved by adding NaOH, was printed into the bath using a pneumatic syringe. The printing process involved a pressure of 60–90 kPa and a speed of 1–7 mm^−1^s, resulting in precise and accurate **P40** structures. The printed structures were then formed by adding 0.1 m HCl to reduce the pH of the **P40** material to a stable level. This process allowed for the successful creation of complex and structurally stable **P40** designs.

### Electrical characterizations

The composite samples were analyzed for ionic conductivity using the PalmSens 4 potentiostat. An AC voltage of 20 mV was applied over a frequency sweep from 100KHz to 10Hz. To prepare the sample, **P40** was pressed and punched with a 10 mm diameter punch. Following that, it was incubated in a petri dish for 24 h, after which it was put between two stainless steel plates. The ionic conductivity, σ, was calculated from the equation

(2) $$\sigma =\frac{\mathrm{Thic}\mathrm{kness}}{\mathrm{Area}\times \mathrm{Rs}}\;$$

where thickness and area were known from the preparation of the sample, while Rs was extracted from the fitted curve to the Nyquist plot using PalmSens software.

For self‐healing studies, a strip of **P40** was connected to an LED powered by four 9 V batteries, and then the strip was cut with a blade. The cut pieces were thereafter put back together in their original position and allowed to self‐heal, while the conductivity of the healed material was monitored and measured.

Tissue conductivity was investigated using the PalmSens V4 Potentiostat, applying 50 mV AC voltage over the frequency range of 1kHz–1MHz. Four groups were studied: muscle after cutting, muscle attached with meat glue, muscle attached with **P40**, and muscle post‐cutting with contact between the two pieces. The goal of the present study was to evaluate **P40** for suitability as a conductive tissue adhesive.

Bending tests on the composite groups were made by clamping them on a fixture so that no pressure or strain would be applied on the connection points. The fixture was designed to be adjustable for a range of angles. During tests, the bending axis was always kept perpendicular to the sensor. All the tests were performed at room temperature. Material impedance was assessed using a 4294A Precision Impedance Analyzer with the operating frequency set at 1 000 Hz.

The gauge factor of the composite material upon 100% applied strain was measured under changes in electrical resistance using a 4294A Precision Impedance Analyzer set at 2 kHz. The general mathematical relationship may be described as

(3) $$\mathrm{Gauge}\;\mathrm{Factor}\;=\frac{\left(\Delta R\;/\;R_{0}\right)\;}{\left(\Delta S\;/\;S_{0}\right)}$$

where ΔR is the change in electrical resistance, R₀ is the initial resistance, ΔS is the applied strain, and S₀ is the initial strain.

In the cyclic tensile tests on **P40** samples, cyclic tensile strain of 50% was applied for 5 000 cycles. During this test, impedance of the samples was continuously tracked using a 4294A Precision Impedance Analyzer working at a frequency of 2 KHz and continuous data capture via LabView software.

### Sensitivity to External Stimuli

The impedance of the composite was characterized in a frequency range of 10–10⁵ Hz under various conditions using Agilent Impedance Analyzer. Pressure sensitivity of the **P40** composite was measured for various forces applied by the Instron machine. The **P40** composite was further subjected to pH 4, 5.5, 7, 8.5, and 10 by dropping different buffer solutions. Humidity and temperature were varied separately inside the humidity chamber in the temperature range 20–70 °C and 40% humidity, while keeping the temperature constant at 37 °C and varying from 25% to 95% humidity.

**P40** Heater Joule heating, the behavior of **P40** heater was studied in DC and AC modes. The dimension size of 20 × 10 × 2 mm with two tin‐plated copper electrodes at both ends as shown in figures were used to construct the heater. The Joule heating behavior of **P40** heater with constant voltages of 200, 230, and 300 V by voltage applied in DC mode was investigated. A sample was recorded and imaged continuously for 60 s interval. For heat power assessment, liquid metal ball of GaIn was used. In case of AC mode, sinusoidal AC voltage with amplitude, a sinusoidal waveform was made available from an amplifier, with a waveform generator. Thermal imaging infrared camera provided temperature monitoring of heater. The temperature was measured by the digital multimeter connected with temperature sensor. Current supplied through the heater was measured by Agilent 34461A digital multimeter.

### Exploration Robotics

In order to conduct snake experiments, six sensors were affixed to a snake toy and connected to a custom‐designed printed circuit board (PCB) to enable the detection of any bending in the robotic snake. This method involved meticulous implementation and calibration, facilitating the successful detection and tracking of the snake toys motion. The robotic snake's movements were recorded by bending and straightening it, while pressure signals were recorded by striking the sensors with a small hammer. The melting of liquid metal was achieved through joule heating with an alternating current (AC) signal, as previously described. Additionally, self‐healing experiments were conducted by intentionally cutting one of the sensors and subsequently healing it. Each of these events was recorded separately, and the data were later consolidated, as illustrated in Figure 4g.

To illustrate the practical application of the concept, **P40** materials were 3D printed around the joints of a plastic snake, showcasing all scenarios in Figure 4h. The actual snake used for the measurements is depicted in Figure S8 (Supporting Information).

### Electrochemical Characterizations

Electrochemical experiments using CV and DPV were performed to analyze hydrogen peroxide (H₂O₂), ascorbic acid (AA), BSA, dopamine, and L‐Dopa. All measurements were carried out using a PalmSens 4 potentiostat (USA) in a three‐electrode system comprising a 3 mm diameter glassy carbon electrode (GCE) modified with **P40**, a platinum wire auxiliary electrode, and an Ag/AgCl reference electrode. For the investigation, **P40** samples with dimensions of 0.5 mm in thickness and 4 mm in diameter were adhered to the GCE, while commercial Zensor electrodes were employed for flexible sensor experiments. The Zensor electrodes, screen‐printed on a polyimide substrate, consisted of a carbon working electrode (1 mm diameter), a carbon counter electrode, and an Ag pseudo‐reference electrode. P40 samples of the same dimensions were adhered to the Zensor electrodes, ensuring complete coverage of the screen‐printed three‐electrode system. Before measurements, the GCE was polished with 0.05 µm alumina slurry, thoroughly rinsed with deionized water, and sonicated sequentially in ethanol and water for 5 min each. Standard solutions of the analytes were prepared in appropriate electrolytes to evaluate the sensing performance of the **P40**‐modified electrodes.

For CV experiments, H₂O₂ and BSA were analyzed in Dulbecco's phosphate‐buffered saline (DPBS), with the **P40**‐modified electrode immersed in 10 mL of DPBS, and stock solution aliquots were added as required. Scans were performed from − 1.0 to + 1.0 V at a scan rate of 50 mV s⁻^1^ over two cycles. Ascorbic acid was analyzed in 0.5 m H₂SO₄ using a similar protocol, but with a potential range of − 0.2 to + 1.0 V at a scan rate of 50 mV s⁻^1^ over two cycles. The second cycle was chosen to demonstrate the CV. DPV experiments for dopamine were conducted in DPBS with an initial potential of − 0.4 V and a final potential of + 1.0 V, using a pulse amplitude of 50 mV, a pulse width of 50 ms, and a scan rate of 20 mV s⁻^1^. For L‐Dopa, flexible Zensor electrodes modified with **P40** were used. Each measurement involved applying 200 µL of analyte solution in 0.5 m H₂SO₄ to the electrode surface, followed by rinsing the electrode with deionized water after each concentration. DPV parameters for L‐Dopa included an initial potential of 0.0 V and a final potential of + 1.0 V, with a pulse amplitude of 50 mV, a pulse width of 50 ms, and a scan rate of 20 mV s⁻^1^.

All experiments were conducted at room temperature (25 ± 2 °C). Electrochemical measurements were performed in triplicate to ensure reliability and reproducibility, and the results were analyzed to evaluate the electrochemical behavior and sensing performance of the **P40**‐modified electrodes.

### Statistical Analysis

GraphPad Prism 10 (GraphPad Software, Inc.) was used to perform statistical analysis. All data are presented as mean  ± standard deviation (SD) with a sample size of at least three. The results are represented in the form of graphs with error bars and shading to show variance. The data was pre‐processed for water content, conductivity, adhesion tests, mechanical tests, and sensing tests to identify outliers. All extreme values were visually assessed and considered outside of a reasonable range. Any data were not excluded unless there were evident measurement errors.

### Ethical Approval and Written Consent

Adhesion properties of the materials in this study were performed using animal by‐products such as skin, heat, muscle, and tendon, with permission from The Danish Veterinary and Food Administration with registration number DK‐13‐oth‐786829 and DK‐123‐oth‐1229243. The flexibility and conformability of the material (Figures S2 and S7, Supporting Information) and electrodes (Figure 5a) were demonstrated by attaching them to a human subject using commercial bandages to display bendability. This study complies with GDPR and ethical regulations, has approval from the Technical University of Denmark (DTU) Research Ethics Officer, and does not involve health applications or human subject testing. Informed written consent was obtained from the participant involved in the research, including consent for using photographic images in the publication.

## Conflict of Interest

The authors declare no conflict of interest.

## Supporting information



Supporting Information

## Data Availability

The data that support the findings of this study are available from the corresponding author upon reasonable request.
