# Supplementary material for: Self‐Maintainable Electronic Materials with Skin‐Like Characteristics Enabled by Graphene‐PEDOT:PSS Fillers
Source: Adv Sci (Weinh). 2025 Apr 25;12(27):2410539. doi: 10.1002/advs.202410539 (PMC12279216; doi:10.1002/advs.202410539)
Supplement: Supplementary file 1 — Supporting Information [file ADVS-12-2410539-s001.docx]

**Supplementary Information**

**Self-Maintainable Electronic Materials with Skin-Like Characteristics Enabled by Graphene-PEDOT:PSS Fillers**

Morteza Alehosseini^1^, Firoz Babu Kadumudi^1,*^, Sinziana Revesz^1^, Parham Karimi Reikandeh^1^, Jonas Rosager Henriksen^1^, Tiberiu-Gabriel Zsurzsan^2^, Jon Spangenberg^3^, Alireza Dolatshahi-Pirouz^1*^

1. Department of Health Technology, Technical University of Denmark, 2800 Kgs. Lyngby, Denmark
2. Department of Electrical and Photonics Engineering, Technical University of Denmark - DTU, Kongens Lyngby, 2800, Denmark
3. Department of Civil and Mechanical Engineering, Technical University of Denmark, 2800 Kgs. Lyngby, Denmark

*Corresponding authors: [fbka@dtu.dk](mailto:fbka@dtu.dk) (Firoz Babu Kadumudi) and [aldo@dtu.dk](mailto:aldo@dtu.dk) (Alireza Dolatshahi-Pirouz)


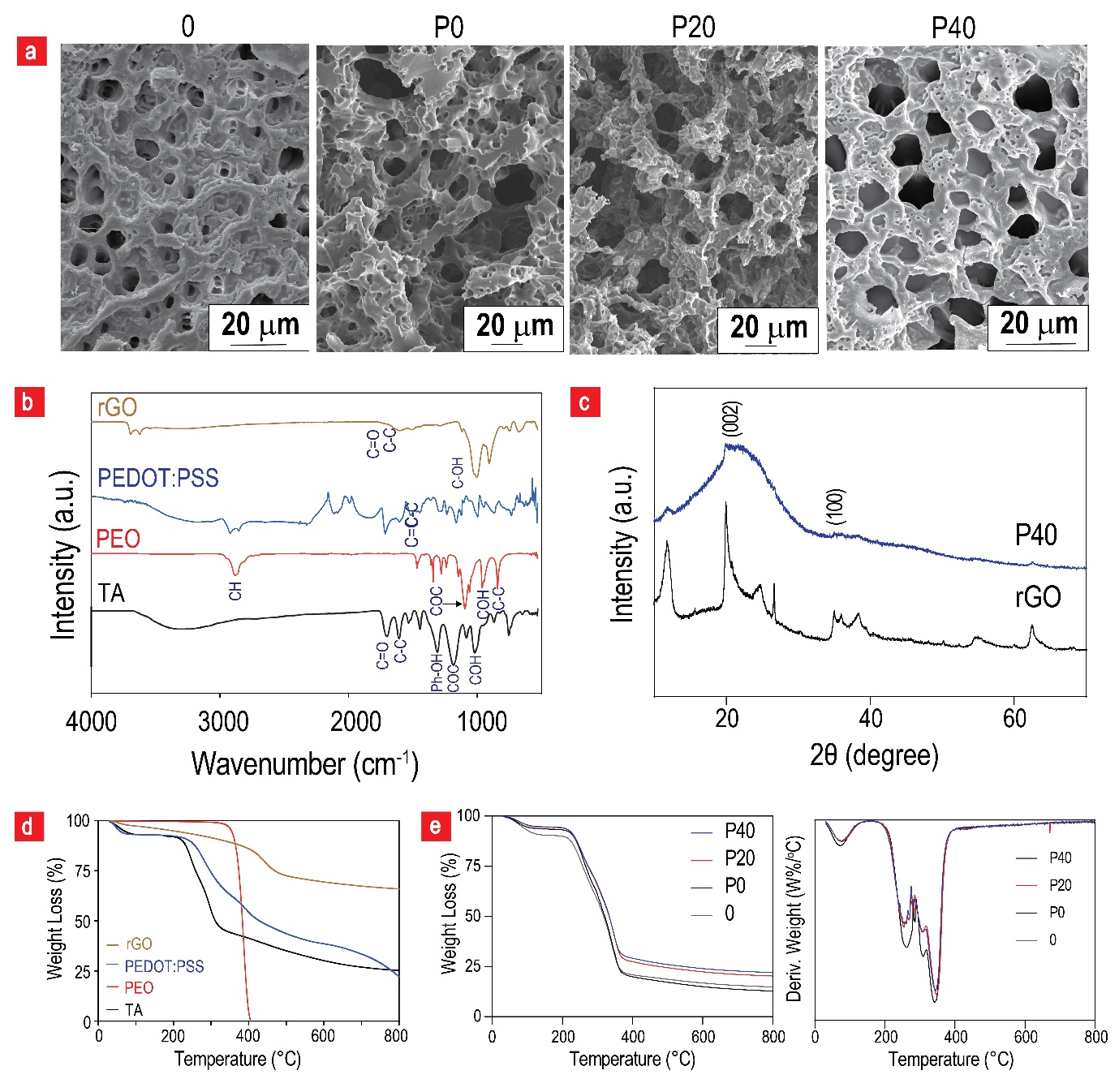


**Figure S1:** (a) Scannin electron microscopic (SEM) images providing microstructural insights of the materials. (b) Fourier-Transform Infrared (FTIR) spectra of pristine samples. (c) X-ray diffraction (XRD) spectra are presented here. Thermogravimetric (TGA) curve of (d) pristine samples and (e) synthesized materials and corresponding derivative thermogravimetric (DTG) curve are presented here. All data are presented as mean, n = 4.

*Chemical characterization:* As shown in Figure S.1.b and Figue 1.c, the characteristic peaks of the PEO segment were identified inside pristine PEO-TA samples corresponding to C-H_2_ stretching at 2880 cm^-1^, C-H bending at 1466 cm^-1^, C-O-C stretching at 1151, 1096, 1061, and 963 cm^-1^, , and CH_2_ wagging at 1363 and 1343.^[1]^ In a similar vein, TA-specific peaks corresponding to OH stretching between 3677-2800 cm^-1^, C=O stretching at 1703 cm^-1^, aromatic C=C at 1610 and 1537 cm^-1^, phenolic C-C at 1445 cm^-1^, phenolic OH, at 1312 cm^-1^, aromatic C-H at 1184 cm^-1^, C-O-C stretching at 1084, -C-H stretching at 953 cm^-1^, and C-H bonds in the benzenes at 869 and 756 cm^-1^.^[2]^ All these distinctive peaks were also seen in the 0% samples, confirming the presence of both TA and PEO components in them. From the **P0** FTIR spectra we further identified shifts for TA from 1703 to 1712 cm^-1^, 1084 cm^-1^ to 1076 cm^-1^, 953 to 943 cm^-1^ corresponding to the C=O stretching, C-O-C peaks and C-OH, respectively. We also identified shifts for PEO from 1096 cm^-1^ to 1076 cm^-1^ and 963 cm^-1^ to 943 cm^-1^ corresponding to C-O-C stretching respectively. The shifts altogether painted a picture of a crosslinking scheme including primarily hydrogen bond interactions such as OH….OH (4.7 kcal mol^-1^), C-O-C….HO (5.02 kcal mol^-1^) and C=O…HO (4.6 kcal mol^-1^) between TA and PEO and between TA and PEO themselves (Figure 1b).^[3, 4]^

The addition of PEDOT:PSS (**P0**) on the other hand did not show the characteristic FTIR peaks of PEDOT at 1520, 1270, and 1057 cm^-1^ corresponding to stretching of C=C, C-C, and C-O-C vibrations, respectively due to the overlap of similar vibrational bands from TA molecules (Figure S1.b and Figue 1.c).^[5, 6]^ However, the observed increase in the peak intensity at 1075, 943, and 842 cm^-1^, corresponding to C-O-C stretching and stretching vibrations of C-S bonds from the thiophene rings of PEDOT, is an indicator of a successful inclusion of PEDOT in the composites.^[6]^ In addition, the shift of the broad –OH peak of TA beginning at 3390 cm^-1^ to 3374 cm^-1^ and the C=O peak of TA at 1712 to 1717 cm^-1^ in **P0** demonstrates the interactions with PEDOT:PSS molecules through OH and C=O groups available on TA and aromatic sulfonate groups present in PEDOT:PSS including the SO_3_H…HO (3-4 kcal mol^-1^) and SO_3_H…O=C hydrogen bonds.^[3, 7]^ The addition of reduced graphene oxide (rGO) on the other hand had no effect on the FTIR peaks of **P0**, a small increase in the intensity of TA was noticed though. This might be due to the interaction between TA-reduced rGO and with the polymeric matrix.

*Thermal degradation studies:* The TGA of pristine samples have been displayed in Figure S1.d. The TGA curves of pure PEO demonstrate a thermal degradation that begins at 330 °C and ends around 405 °C. This is most likely due to the evaporation of -C-O- and -C-C- bonds of the polymer chain through the formation of CO_X_H_Y_ compounds and the release of CO, CO_2_ and H_2_O.^[8]^ Pristine PEDOT:PSS powder showed an initial weight loss up to 200 °C due to the loss of water, and a fast weight loss over 250 °C due to the breakdown of PSS via the rupture of it’s sulfonate group at temperatures above 200 °C. A third weight loss event at around 600 °C in the PEDOT-PSS curve is most likely due to the rupture of it’s entire polymeric backbone. The TGA curve of TA on the other hand showed a two-step degradation process consisting of an initial weight loss up to 200 °C due to the loss of water and degradation of it’s short hydroxyl groups. This was followed by breakdown of the gallic acid units and glucose units after 200 °C. Similarly, rGO exhibited a linear drop in weight till 400 °C due to the decomposition of the TA attached to rGO. The presence of epoxy oxygen molecules on the partly reduced rGO surface caused a 15% drop in temperature between 400 and 500 °C.

The TGA analysis of the composites (Figure S1.e) as firstly analyzed through a DTG analysis (Figure S1.f). This analysis revealed a thermal loss event below 200 ^o^C due to water loss and three events over 200 ^o^C for all composites due to polymeric degradation. Of these three events, the first two at 253 and 305 ^o^C corresponds to TA, whereas the highest peak at 354 ^o^C corresponds to PEO. Besides gaining a deeper understanding of the molecular interactions in the composites, and their associated strengths, we can also use TGA measurements in Figure S1.e-f for probing the approximate fraction of each compound within the composites. We can do this by comparing the residual weights at 800 °C of pure polymer/filler and composites. For instance for the 0% composite, we can see that the residual weight at 800 °C was 15 %, since PEO has 0% residual weight at 800 °C, whereas TA powder shows 25% residual weight at this temperature, this is in accordance with a composition comprising 60 weight% TA and 40 weight% PEO. Due to the competitive interaction between PEDOT:-PSS and TA with PEO molecules, the addition of PEDOT:PSS reduced this residual weight to 12 %, indicating a lower concentration of TA in **P0** samples compared to the pristine samples. Increasing the concentration of rGO resulted in a higher residual weight at 800 °C due to the higher residual weight of the pristine rGO (66%).

Crystallinity studies: All the DSC associated results have been displayed in Figure 1.d. In the case of PEO, an endothermic peak at 67 °C corresponding to it’s melting temperature (Tm) and close to the literature values at 65-67 °C was found.^[9]^ The calculated melting enthalpy (ΔHm) for this peak was determined to be 125 J/g, corresponding to 58% crystallinity in the pure PEO powder. It is worth noting that PEO's crystallinity typically ranges from 60-95% in the literature, depending on its molecular weight. TA on the other hand exhibited a broad peak starting at 81 °C and ending at 150 °C, which corresponded to the evaporation of moisture and bound water within its super hydrophilic structure. When PEO and TA were combined, the Tm of PEO was significantly altered, resulting in a non-crystalline wide peak spanning from 68 °C to 147 °C. This behavior was diminished in the second run, indicating that the peak corresponded to the water loss temperature (Tw) of TA caused by the loss of water.


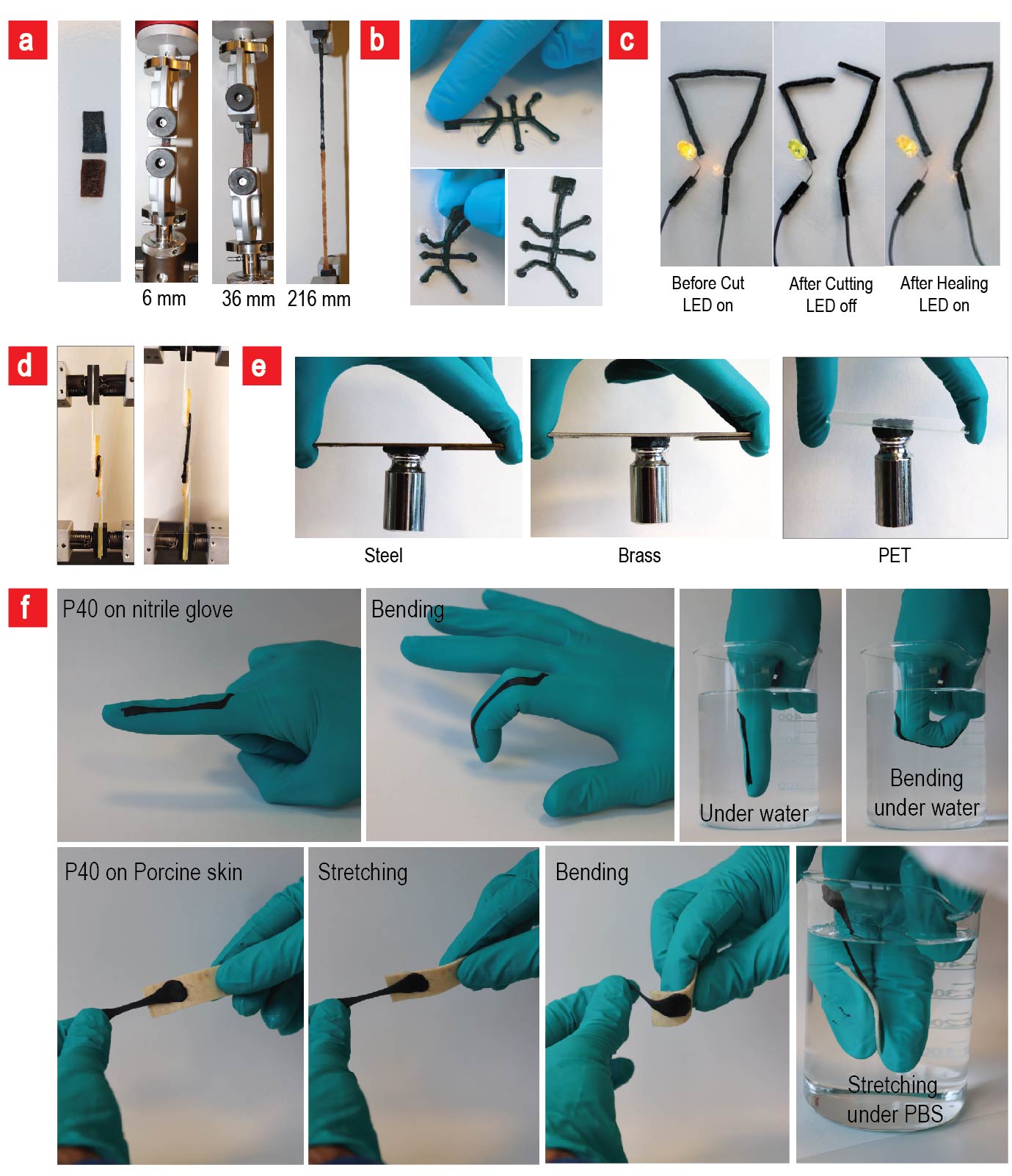


**Figure S2: Self-healing, stretchability, moldability, and adhesion. (**a) Photographic images illustrating the extraordinary stretchability of **P40**, reaching up to 36 times its original length without damage after self-healing. (b) Highlighting the exceptional moldability of **P40** in the Teflon mold-casted complex structure. (c) **P40** demonstrates its high moldability in a hand-built self-healing electronic circuit connected to an LED lamp. (d) The lap shear test on pig skin. (e) Photographic images demonstrating the adhesion property of **P40** on various surfaces (steel, brass, PET). (f) Photographic images demonstrate that **P40** can adhere to nitrile gloves and porcine skin, maintaining stability during dynamic movements and even when submerged in water or PBS.

*Self-healing capacity:* The images in Figure S2.a are a clear testimony of the amazing self-healing capacity of our nanocomposite materials. Here, we used samples with either a yellow or black color, so we could distinguish them from one another. The individual samples were then joined together to begin the healing process – something that only lasted a few minutes. Notably, after the healing process the new pieces could stretch up to 36 times their original length without being damaged (Figure S2.a). It also displayed great moldability – even after self-healing as shown in Figure S2.b).

*Adhesion:* Initially, we employed a simple gravity-based method, in which the materials were sandwiched between a 100 g weight and surface in interest, after which the weight was put into free-fall mode (Figure S2.e). The images obtained in the preliminary adhesion experiments demonstrated a strong adhesion between the composites and Brass, Steel and polyethylene terephthalate (PET).


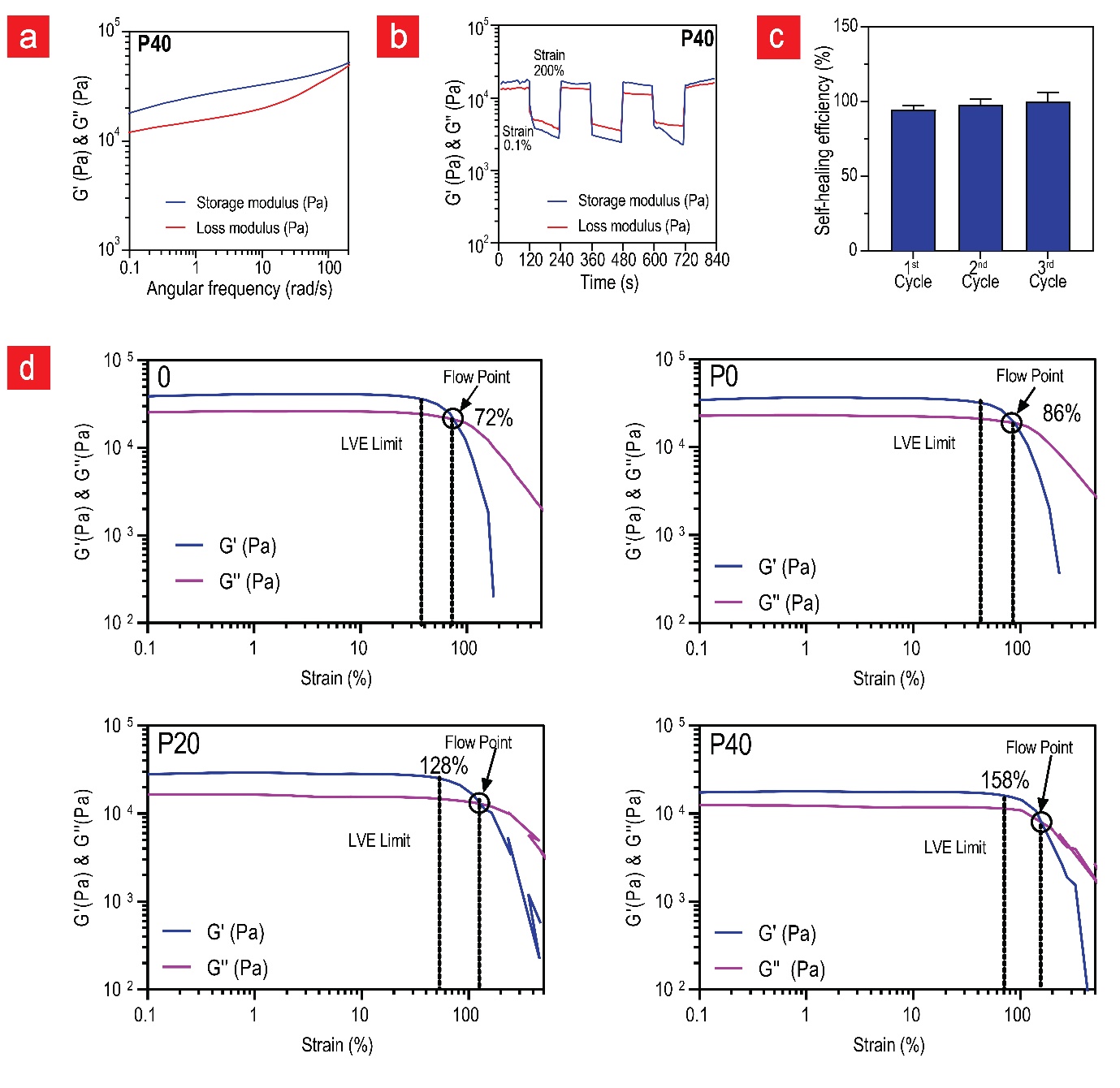


**Figure S3:** a) Dynamic frequency sweep data for **P40**. b) Cyclic shear-straining tests at 10 rad/s and 200% strain, assessing the effectiveness of the self-healing capacity of **P40**. c) The calculated self-healing efficiency from the cyclic shear-straining tests for up to 3 cycles are show here. d) Rheology test to define the LVE region and breakage point of the respective samples **0**, **P0**, **P20**, and **P40.** All data are presented as mean ± SD, n = 4.

Rheology: To deepen our understanding of the mechanical properties of the different composites we turned to rheology. We started with a frequency sweep with frequencies ranging from 0.1 rad/s to 100 rad/s and found that the composites behaved solid-like all the way in accordance with their gum-like consistency, since the storage modulus was higher than the loss modulus (Figure S3.a). We therefore choose 10 rad/s for the follow up rheology experiments, as this value was within the viscoelastic range that is of interest for us and not the liquid one. From the strain-sweep measurements in Figure S3.d we determined the linear viscoelastic region to < 100 % strain for all variants, which corresponds to an undisturbed state. Around 120 % both the loss modulus and storage began to decrease marking a transformation into a more disturbed state. After 158% strain the loss and storage modulus intersected for **P40** – something which in theory only happens when the material is destroyed. As the graphene concentration was lowered the disturbance strain point dropped from 158 % to 72 %. Based on Figure S3.a and Figure S3.d we thus decided to perform cyclic shear-straining tests at 10 rad/s and 200 % strain to see how fast and how well the **P40** healed after complete damage (Figure 3.g). From here we found that the self-healing time was 2 minutes with a self-healing efficiency that remained close to 100 % even after three healing cycles (Figure S3.b-c).

**
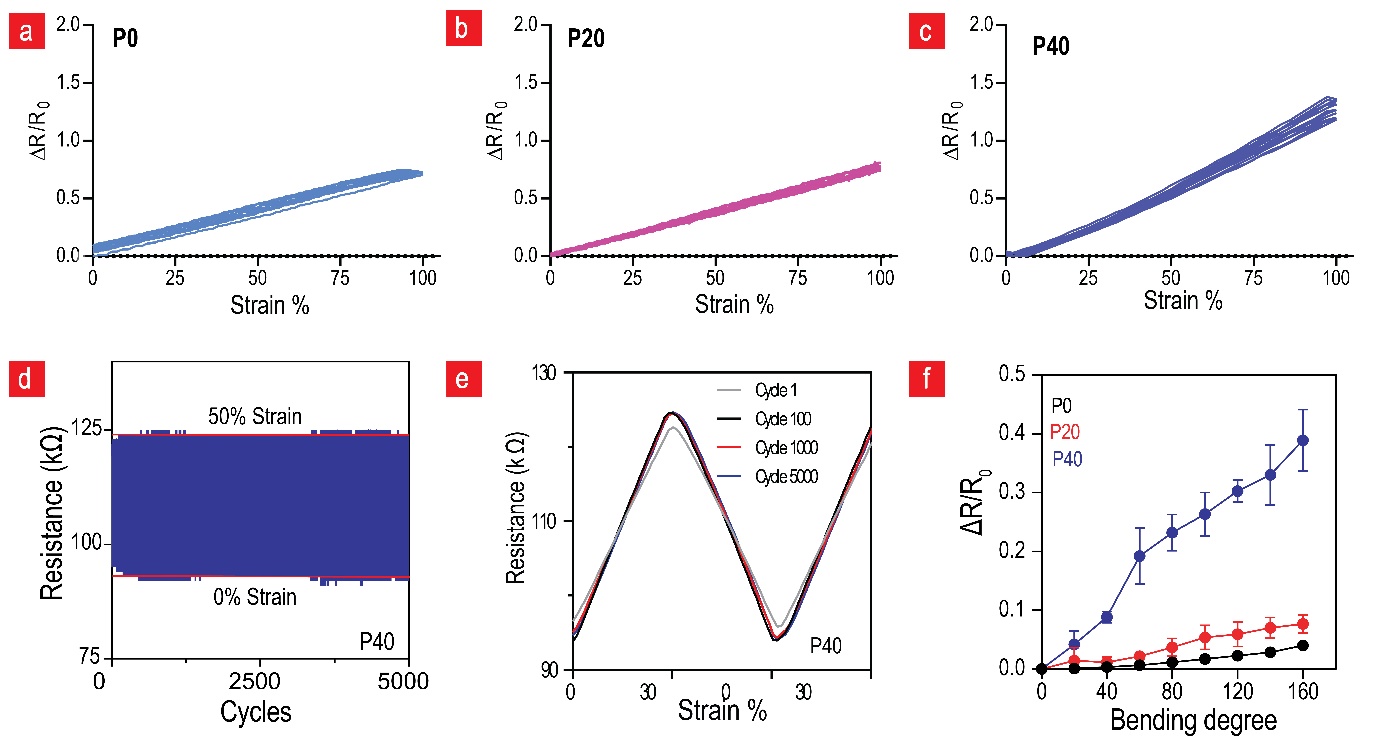
**

**Figure S4:** Cyclic strain test of the different composites (a) **P0**, (b) **P20**, (c) **P40**, and (d) comparison of different cycles of **P40.** d) Cyclic tensile strain of up to 50% was applied to P40 for 5000 cycles, and e) shows enlarged views of the impedance values after 1, 100, 1000, and 5000 cycles. f) This panel shows the bending angle and corresponding relative resistance changes in the samples (n = 3). All data are presented as mean ± SD.

*Motion and bending sensing:* We found a sharp relative resistance (ΔR/R_0_, with ΔR = R - R_0_) increase after straining the samples up to 100 %– this increase was largest for **P40** (Figure S4.a-c). This slope is in layman terms referred to as the Gauge Factor (GF) and a measure of strain sensitivity. Therefore, the higher the GF-factor the smaller material strains can be detected. The Gauge factor increased almost two-fold from 0.66 ± 0.04 to 1.3 ± 0.15 after incorporating 40 % GO into the composites (**P40**) (Figure 3.c). This is arguably due to a weak GO percolation network inside the composite matrix mediated by hydrogen bonds, which can give rise to the observed sharp conductivity increase, but at the same time they are easy to short circuit via mechanical force. We saw a similar trend after bending the samples (Figure S4.f). Even still, the electrical connections are sufficiently robust to enable the material to be deformed 5000 times without permanently damaging the electrical properties (Figure S4.b). This is most likely facilitated by the rapid electrical self-healing observed in Figure 1 and S2. In the domains of wearable electronics, digital skin devices, and soft robotics, there is a significant demand for digital items that possess such cyclic stretchability and durability. As you will bear witness two in the following sections our nanomaterials are also sensitive to bending and strain (motion), due to an inherent link present in them between resistance and strain. Finally, due to motion sensitivity our skin electronics can therefore not only be used to mend electroactive muscles, but also to monitor their flexing and straining afterwards to gain a better understanding of the healing process. Obviously, muscle tissue that has not mended properly will display impaired motion during activity than it’s fully repaired counterpart.


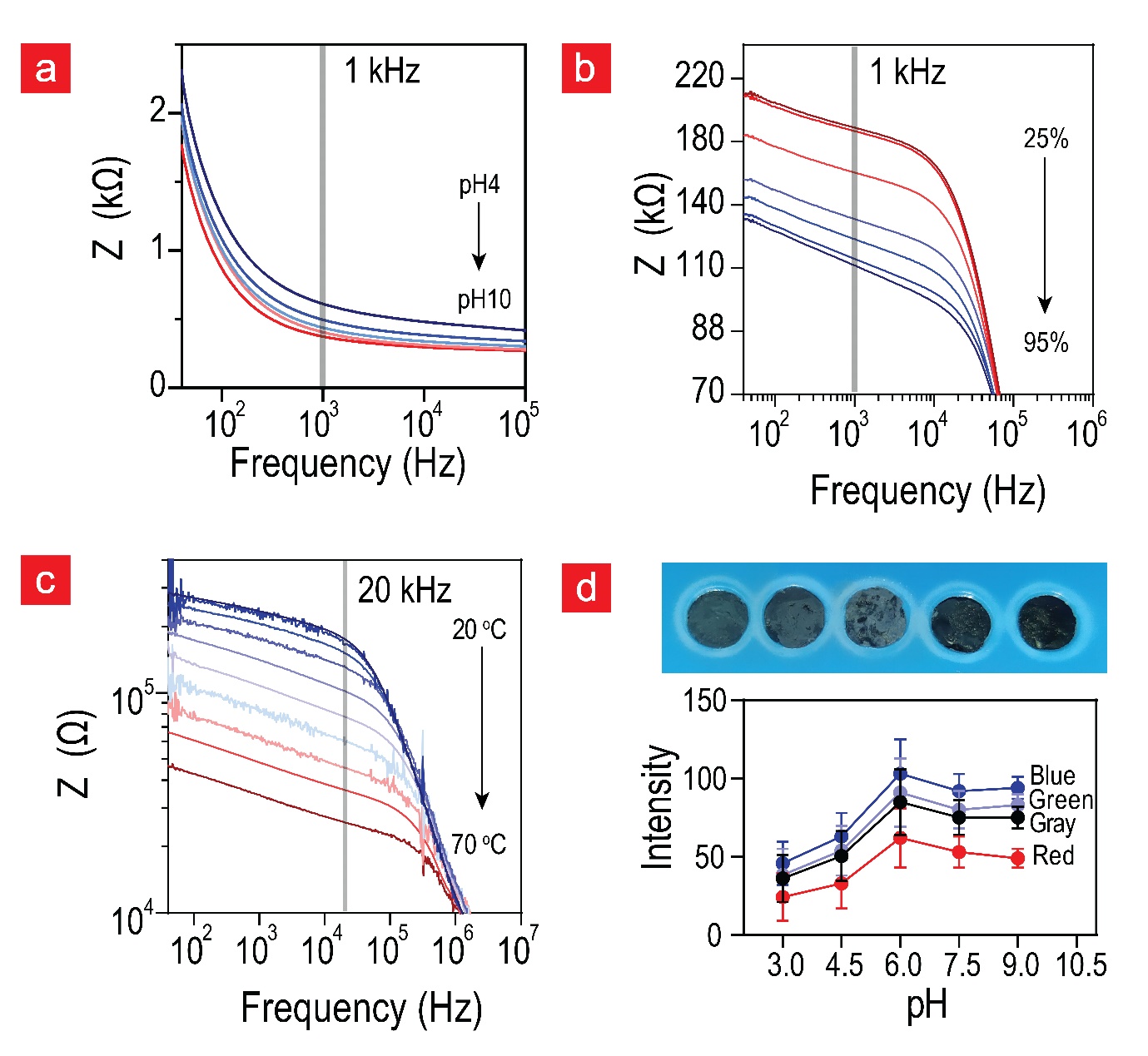


**Figure S5:** Detailed electrical impedance characterization of **P40's** physical sensing towards (a) pH, (b) humidity, and (c) temperature. d) Gradual color transformation from grayish to black as pH-value increased from 3.0 to 9.0. All data are presented as mean ± SD, n = 4.

*pH, Humidity and Temperature sensing:* In Figure S5.a-c we measured the impedance of the materials, while changing the pH (Figure S5.a), humidity (Figure S5.b) and temperature (Figure S5.c). In detail, the impedance of **P40** (Figure S5.a) at an applied voltage of 50 mV, showed a marginal variation as function of pH-value at low frequencies (<100 Hz), while the variation was much larger at higher frequencies (>100Hz), which prompted us to use data obtained at 1 kHz to depict the pH-sensitivity. In contrast to pH, the impedance of **P40** at various humidity’s (Figure S5.b) and temperatures (Figure S5.c)) showed a reverse trend with a very small functional variation at higher frequencies (>10kHz) compared with low frequencies (< 10kHz). We therefore decided to use 1 kHz and 20 kHz for depicting the humidity and temperature sensitivity to assure the best possible signal fidelity.


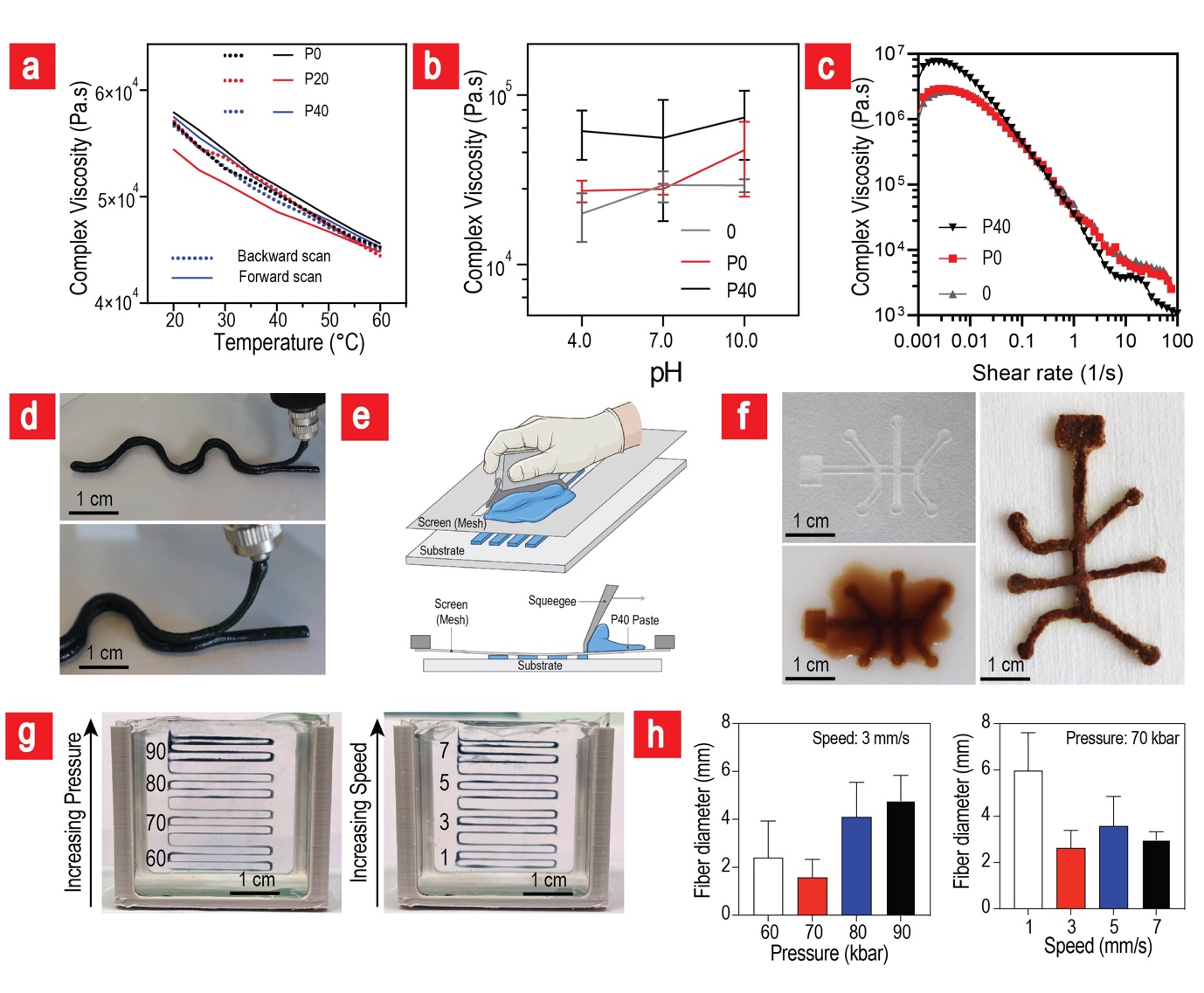


**Figure S6: 3D printing.** a) Evaluation of temperature-dependent changes in the complex viscosity of the materials b) Complex viscosity at different pH levels and c) viscosity behavior as function of shear rate. d) Harnessing the observed shear-thinning property of **P40** for extrusion printing. e) An illustration depicting the screen-printing technique, while panel f) shows how we could create complex electrical circuits by utilizing the screen-printing method. g-h) Here, we have utilized a suspension bath for 3D printing complex structures as well as optimizing print resolution with different extrusion pressures and speeds. All data are presented as mean ± SD, n = 4.

*3D extrusion and screen printing:* From Figure S6.a, we could see that as the temperature increased from 20 to 60 °C a decrease in complex viscosity from 60,000 to 45,000 Pa.s occurred. What's even more fascinating is that this decrease was reversible when the sample was cooled back down to 20 °C. This suggests a temperature-dependent reversible behavior. Additionally, we found that the viscosity decline as a function of temperature was independent of the graphene oxide (GO) content. This indicates that the viscosity change is primarily influenced by temperature rather than the GO content (Figure S6.a). Furthermore, we were able to manipulate the viscosity by adjusting the pH value. Lowering the pH from 10 to 4 resulted in a decrease in viscosity (Figure S6.b). Not only does **P40** boast a straightforward and non-toxic degradation pathway that operates based on pH and temperature, but this property also offers the added benefit of on-demand recycling or degradation. For instance, this feature can assist in reducing electronic waste generation and in preserving the environment by keeping it safe from potential man-made pollution. Moreover, our findings reveal that the composites exhibit shear-thinning behavior. For instance, by increasing the shear rate to 100 /s, we could observe a transition from a solid-like phase to a more liquid-like one (Figure S6.c). This is like what a liquid being extruded from a needle typically experiences. In summary, our rheological assays demonstrate different means for us to tap into too readily 3D extrude the **P40** variant into complicated 3D architectures. In Figure S6.d, we have used the shear-thinning property of the **P40** variant to extrude spiral-like structure with a handheld device. In Figure S6.e-f we have instead tapped into screen printing and used the temperature responsiveness of the **P40** variant to change it into a more flowable phase that could readily penetrate the screen mask, and thereby transfer the mask pattern onto the substrate.

*Support bath printing:* In Figure S6.g we have used a laponite-based medium (3 wt. %/v + viscosity 17 Pa.s) for this purpose – and we have tried to optimize the print resolution by using different extrusion pressures and speed values. The fiber diameters retrieved from the prints were later analyzed and depicted in Figure S6.h. From here it is clearly seen that the smallest achievable diameters were in the range 1 mm – 2 mm corresponding to 70 kbar extrusion pressure and 3 mm/s print speeds.

*Freeze-dried* ***P40*** inks: In another elegant 3D printing approach (Figure S7), we first freeze-dried the **P40** variant and then crushed it into a soluble powder form. We subsequently stored it in the fridge for over one month in a conventional glass vial, after which it was dissolved in PBS and extruded into a complex electrical circuit capable of lighting a LED lamp. The procedure was quite simple and thus easy for other laboratories to adapt and implement. The powdered ink was then dissolved and printed on a small bandage. We then demonstrated that it could be used to monitor the motion of a wrist due to the resistance increase associated with mechanical strains over the printed circuit. This methodology holds more promise than the others, as it simultaneously presents an economically efficient route for further down-stream applications and possible commercialization options.


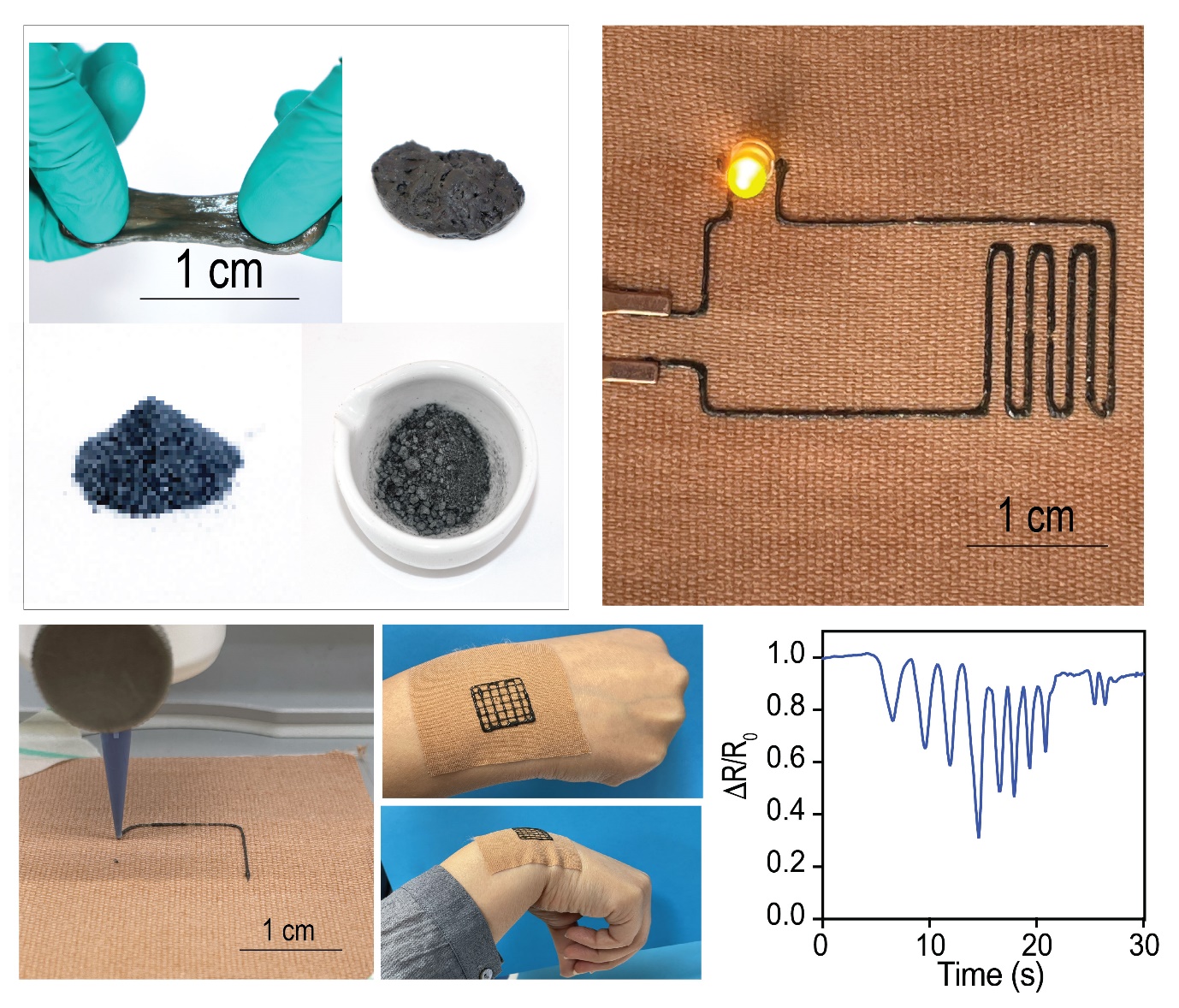


**Figure S7:** 3D printing of **P40** by freeze drying it and then re-thawing it prior to the extrusion printing process.


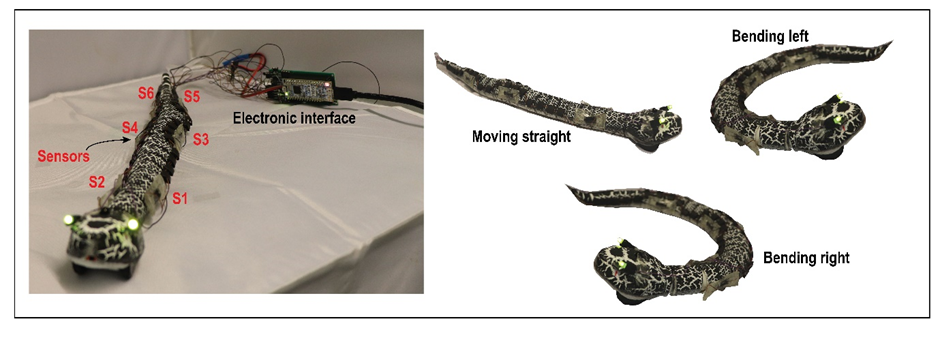


**Figure S8:** The actual robotic snake used in the measurements displayed in Figure 4.


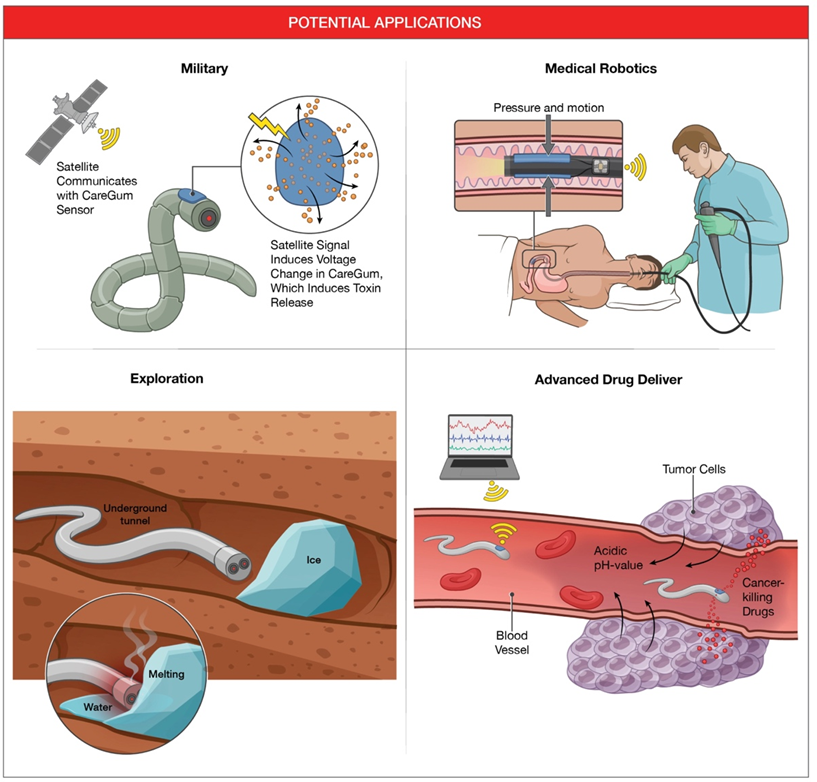


**Figure S9:** Illustration showcasing possible applications in military, exploratory and medical robotics.


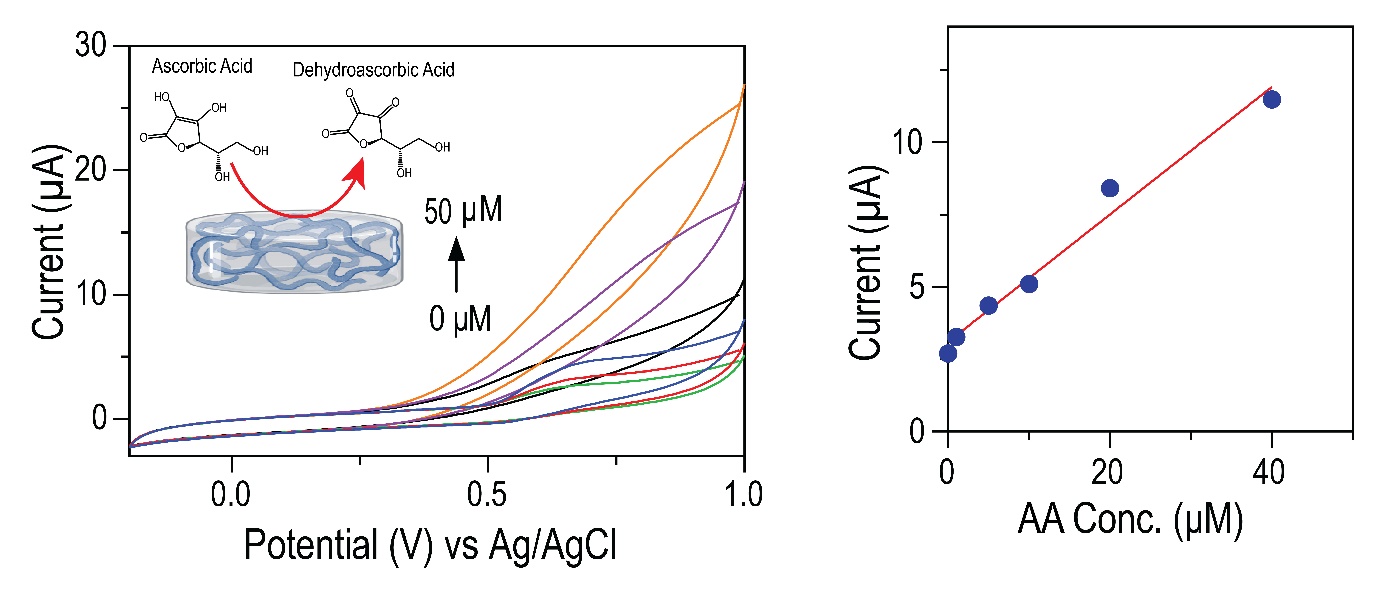


**Figure S10:** Electrochemical sensing of Ascorbic acid using **P40.** All data are presented as mean, n = 4.


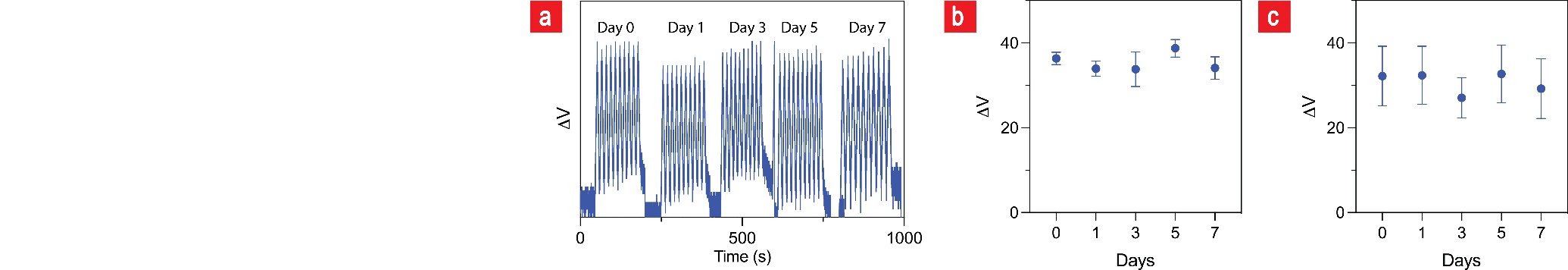


**Figure S11: Stability studies of P40 samples over 1 week.** (a) Cyclic tensile strain of up to 20% applied to P40 for 10 cycles across 7 days. (b) Average voltage signal change for a single sample across different cycles on different days. (c) Average voltage signal change for five samples across different cycles on different days**.**

**Table S1.** Self-healing time, young's modulus, and strain recovery data of the literature values of hydrogels with both adhesive and self-healing characteristics published in recent years.

| **S.No.** | **Elastomer** | **Self-Healing Time (minutes)** | **Youngs Modulus (kPa)** | **Strain Recovery (%)** | **Ref.** |
| --- | --- | --- | --- | --- | --- |
| 1 | PAA/zwitterion elastomers | 720 | 80 | 800 | ^[10]^ |
| 2 | PAA/PEDOT:PSS | 120 | 8 | 200 | ^[11]^ |
| 3 | PAAm/PAA-Fe3+/NaCl | 1440 | 490 | 200 | ^[12]^ |
| 4 | PAA-co-PAAS@ohPEI@TTpy hydrogel | 1 | 60 | 100 | ^[13]^ |
| 5 | PAA-PDA/CNT Hydrogel | 1440 | 200 | 400 | ^[14]^ |
| 6 | P(BMA-co-MEA) Liquid-Free ICEs | 1440 | 670 | 200 | ^[15]^ |
| 7 | PAA/[EMIM][DEP]/HPC (PIH) ionogels | 1440 | 216 | 200 | ^[16]^ |
| 8 | PAM/PBA-IL/CNF Hydrogels | 150 | 384 | 1000 | ^[17]^ |
| 9 | P(DAC-co-AM)/CNT composite hydrogel | 1440 | 200 | 500 | ^[18]^ |
| 10 | PVA/PAA-PAM-IS/GC Composite Hydrogels | 720 | 100 | 100 | ^[19]^ |
| 11 | PDMA ion-conductive gel | 1 | 100 | 200 | ^[20]^ |
| 12 | Acrylamide-PVP | 1 | 366 | 600 | ^[21]^ |
| 13 | Gel–OSA–PVA | 1440 | 92 | 400 | ^[22]^ |
| 14 | PVA-SA-DOPA-AgNW-borax Hydrogel | 1 | 1000 | 200 | ^[23]^ |
| 15 | PVA/ MXene-Ag-Sucrose | 5 | 150 | 50 | ^[24]^ |
| 16 | PVA / AgNP/DACNF-X/TA | 1 | 114 | 300 | ^[25]^ |
| 17 | PVA-PAA-TA | 5 | 50 | 30 | ^[26]^ |
| 18 | PVA-PAA-TA-fCNT | 5 | 100 | 100 | ^[27]^ |
| 19 | PEG-Based Double-Network Hydrogels | 1 | 8.2 | 800 | ^[28]^ |
| 20 | UPy-CPU elastomer | 1440 | 1490 | 1000 | ^[29]^ |
| 21 | PF4M6 | 30 | 1120 | 50 | ^[30]^ |
| 22 | γ-PGA/PEDOT:PSS conductive hydrogels | 720 | 75 | 300 | ^[31]^ |
| 23 | PEDOT/EG/TA | 1 | 450 | 30 | ^[32]^ |
| 24 | Agarose-Zwitterion Hydrogel | 20 | 528 | 600 | ^[33]^ |
| 25 | Tannic acid-encapsulated cellulose nanocrystals | 720 | 498 | 700 | ^[34]^ |
| 26 | Ti3C2Tx MXene-based PDMAEA-Q/CS hydrogels | 60 | 100 | 100 | ^[35]^ |
| 27 | **P40** | 1 | 600 | 600 | This work |

**References**

[1] I. Pucić, T. Jurkin, *Radiation Physics and Chemistry* **2012**, 81, 1426; E. A. Ozturk, Z. R. Ege, S. Murat, G. Erdemir, S. Kuruca, Z. E. Erkmen, O. Duygulu, O. Gunduz, T. Caykara, M. S. Eroglu, *International Journal of Biological Macromolecules* **2022**, 217, 562; Y.-l. Su, J. Wang, H.-z. Liu, *Macromolecules* **2002**, 35, 6426.

[2] P. Erdem, E. A. Bursali, M. Yurdakoc, *Environmental Progress & Sustainable Energy* **2013**, 32, 1036; A. Ricci, K. J. Olejar, G. P. Parpinello, P. A. Kilmartin, A. Versari, *Applied Spectroscopy Reviews* **2015**, 50, 407.

[3] T. Steiner, *Angewandte Chemie-International Edition* **2002**, 41, 48; D. F. Lewis, *TheScientificWorldJOURNAL* **2004**, 4, 1074.

[4] D. Wang, D. Wang, C. Deng, K. Wang, X. Tan, Q. Liu, *Chemical Engineering Journal* **2022**, 446, 137403.

[5] J. L. Carter, C. A. Kelly, M. J. Jenkins, *Polymer Journal* **2023**, 55, 253.

[6] C. Yeon, G. Kim, J. Lim, S. Yun, *RSC advances* **2017**, 7, 5888.

[7] J. Cao, X. Yang, J. Rao, A. Mitriashkin, X. Fan, R. Chen, H. Cheng, X. Wang, J. Goh, H. L. Leo, *ACS Applied Materials & Interfaces* **2022**, 14, 39159.

[8] M. Jakić, N. S. Vrandečić, I. Klarić, *Polymer degradation and stability* **2013**, 98, 1738; K. Pielichowski, K. Flejtuch, *Journal of Analytical and Applied Pyrolysis* **2005**, 73, 131.

[9] P. Maitra, J. Ding, H. Huang, S. L. Wunder, *Langmuir* **2003**, 19, 8994; O. Omosola, D. M. Chipara, M. Uddin, K. Lozano, M. Alcoutlabi, V. Padilla, M. Chipara, *Journal of Applied Polymer Science* **2022**, 139, 52055.

[10] W. Zhang, B. Wu, S. Sun, P. Wu, *Nature Communications* **2021**, 12, 4082.

[11] Q. Gao, C. Li, M. Wang, J. Zhu, C. Gao, *Journal of Materials Chemistry C* **2023**, 11, 9355.

[12] S. Li, H. Pan, Y. Wang, J. Sun, *Journal of Materials Chemistry A* **2020**, 8, 3667.

[13] C. Chen, X. Pang, Y. Li, X. Yu, *Small* **2024**, 20, 2305875.

[14] J.-J. Wang, Q. Zhang, X.-X. Ji, L.-B. Liu, *Chinese Journal of Polymer Science* **2020**, 38, 1221.

[15] C. Luo, Y. Chen, Z. Huang, M. Fu, W. Ou, T. Huang, K. Yue, *Advanced Functional Materials* **2023**, 33, 2304486.

[16] Y. Zhou, L. Wang, Y. Liu, X. Luo, Y. He, Y. Niu, Q. Xu, *Chemical Engineering Journal* **2024**, 484, 149632.

[17] X. Yao, S. Zhang, L. Qian, N. Wei, V. Nica, S. Coseri, F. Han, *Advanced Functional Materials* **2022**, 32, 2204565.

[18] C. Pan, J. Wang, X. Ji, L. Liu, *Journal of Materials Chemistry C* **2020**, 8, 1933.

[19] J. Min, Z. Zhou, J. Zheng, C. Yan, H. Sha, M. Hong, H. Fu, *Macromolecular Materials and Engineering* **2022**, 307, 2100948.

[20] L. Wang, S. Liu, J. Cheng, Y. Peng, F. Meng, Z. Wu, H. Chen, *Soft Matter* **2022**, 18, 6115.

[21] Z. Guo, H. Gu, Y. He, Y. Zhang, W. Xu, J. Zhang, Y. Liu, L. Xiong, A. Chen, Y. Feng, *Chemical Engineering Journal* **2020**, 388, 124282.

[22] K. Li, J. Wang, P. Li, Y. Fan, *Journal of Materials Chemistry B* **2020**, 8, 4660.

[23] L. Fan, L. Hu, J. Xie, Z. He, Y. Zheng, D. Wei, D. Yao, F. Su, *Biomaterials Science* **2021**, 9, 5884.

[24] C. Li, A. Zheng, J. Zhou, W. Huang, Y. Zhang, J. Han, L. Cao, D. Yang, *New Journal of Chemistry* **2023**, 47, 6621.

[25] D. Fu, R. Yang, R. Wang, Y. Wang, Y. Li, H. Bian, *Carbohydrate Polymers* **2024**, 334, 122060.

[26] J. Park, T. Y. Kim, Y. Kim, S. An, K. S. Kim, M. Kang, S. A. Kim, J. Kim, J. Lee, S. W. Cho, *Advanced Science* **2023**, 10, 2303651.

[27] J. Park, J. Y. Kim, J. H. Heo, Y. Kim, S. A. Kim, K. Park, Y. Lee, Y. Jin, S. R. Shin, D. W. Kim, *Advanced Science* **2023**, 10, 2207237.

[28] K. Chen, Y. Feng, Y. Zhang, L. Yu, X. Hao, F. Shao, Z. Dou, C. An, Z. Zhuang, Y. Luo, *ACS applied materials & interfaces* **2019**, 11, 36458.

[29] M. W. M. Tan, G. Thangavel, P. S. Lee, *Advanced Functional Materials* **2021**, 31, 2103097.

[30] P. Hu, Y. Zhang, S. Zhou, T. Chen, D. Wang, T. Liu, Y. Wang, J. Chen, Z. Wang, J. Xu, *Chemical Engineering Journal* **2023**, 464, 142543.

[31] C. Zhang, M. Wang, C. Jiang, P. Zhu, B. Sun, Q. Gao, C. Gao, R. Liu, *Nano Energy* **2022**, 95, 106991.

[32] X. Zhou, P. Kateb, J. Fan, J. Kim, G. A. Lodygensky, B. Amilhon, D. Pasini, F. Cicoira, *Journal of Materials Chemistry C* **2024**, 12, 5708.

[33] J. Liu, Y. Zhou, J. Lu, R. Cai, T. Zhao, Y. Chen, M. Zhang, X. Lu, Y. Chen, *Chemical Engineering Journal* **2023**, 475, 146340.

[34] X. Zhang, Q. Fu, Y. Wang, H. Zhao, S. Hao, C. Ma, F. Xu, J. Yang, *Advanced Functional Materials* **2024**, 34, 2307400.

[35] Y. He, Z. Deng, Y.-J. Wang, Y. Zhao, L. Chen, *Carbohydrate Polymers* **2022**, 291, 119572.
